# Invitation methods for Indigenous New Zealand Māori in lung cancer screening: Protocol for a pragmatic cluster randomized controlled trial

**DOI:** 10.1371/journal.pone.0281420

**Published:** 2023-08-01

**Authors:** Kate Parker, Sarah Colhoun, Karen Bartholomew, Peter Sandiford, Chris Lewis, David Milne, Mark McKeage, Rawiri McKree Jansen, Kwun M. Fong, Henry Marshall, Martin Tammemägi, Nicole M. Rankin, Sandra Hotu, Robert Young, Raewyn Hopkins, Natalie Walker, Rachel Brown, Sue Crengle

**Affiliations:** 1 Planning Funding and Outcomes, Waitematā District, Te Whatu Ora and Te Toka Tumai Auckland District, Te Whatu Ora, Auckland, New Zealand; 2 Ngāi Tahu Māori Health Research Unit, School of Health Sciences, University of Otago, Dunedin, New Zealand; 3 Waitematā District, Te Whatu Ora, Auckland, New Zealand; 4 Te Toka Tumai Auckland District, Te Whatu Ora, Auckland, New Zealand; 5 University of Auckland, Auckland, New Zealand; 6 Te Aka Whai Ora, Manukau, New Zealand; 7 National Hauora Coalition, Auckland, New Zealand; 8 Department of Thoracic Medicine, Prince Charles Hospital, Brisbane, Queensland, Australia; 9 University of Queensland Thoracic Research Centre, Brisbane, Queensland, Australia; 10 Brock University, St. Catharines, Ontario, Canada; 11 Centre for Health Policy, Melbourne School of Population and Global Health, University of Melbourne, Melbourne, Australia; 12 Sydney School of Public Health, University of Sydney, Camperdown, Australia; UNITED KINGDOM

## Abstract

Lung cancer screening can significantly reduce mortality from lung cancer. Further evidence about how to optimize lung cancer screening for specific populations, including Aotearoa New Zealand (NZ)’s Indigenous Māori (who experience disproportionately higher rates of lung cancer), is needed to ensure it is equitable. This community-based, pragmatic cluster randomized trial aims to determine whether a lung cancer screening invitation from a patient’s primary care physician, compared to from a centralized screening service, will optimize screening uptake for Māori. Participating primary care practices (clinics) in Auckland, Aotearoa NZ will be randomized to either the primary care-led or centralized service for delivery of the screening invitation. Clinic patients who meet the following criteria will be eligible: Māori; aged 55–74 years; enrolled in participating clinics in the region; ever-smokers; and have at least a 2% risk of developing lung cancer within six years (determined using the PLCO_M2012_ risk prediction model). Eligible patients who respond positively to the invitation will undertake shared decision-making with a nurse about undergoing a low dose CT scan (LDCT) and an assessment for Chronic Obstructive Pulmonary Disease (COPD). The primary outcomes are: 1) the proportion of eligible population who complete a risk assessment and 2) the proportion of people eligible for a CT scan who complete the CT scan. Secondary outcomes include evaluating the contextual factors needed to inform the screening process, such as including assessment for Chronic Obstructive Pulmonary Disease (COPD). We will also use the RE-AIM framework to evaluate specific implementation factors. This study is a world-first, Indigenous-led lung cancer screening trial for Māori participants. The study will provide policy-relevant information on a key policy parameter, invitation method. In addition, the trial includes a nested analysis of COPD in the screened Indigenous population, and it provides baseline (T0 screen round) data using RE-AIM implementation outcomes.

## Introduction

Globally, lung cancer (LC) is the leading cause of cancer death [1]. In Aotearoa New Zealand (NZ), it is a common cause of cancer deaths for both the Indigenous Māori and the non-Māori populations [2,3]. There are significant ethnic inequities in incidence and mortality [3–8], with Māori women experiencing four times the rate of lung cancer compared to non-Māori women and Māori men having a rate that is nearly three times higher than non-Māori men [3–8]. Māori develop lung cancer around six years earlier than non-Māori, and at lower smoking exposures [8,9]. Māori mortality rates from lung cancer are 30% higher than for non-Māori [3], due to a range of factors including treatment pathway delays, access barriers and systemic racism [10,11], later diagnosis and comorbidities [5]. Although tobacco use in adults aged ≥15 years has been reducing over time in Aotearoa NZ, use is much higher in Māori (26% in 2020/21, vs 20% Pacific, 6% Asian, 9% NZ European) [12], and is the leading modifiable risk factor for both LC and chronic obstructive pulmonary disease (COPD) [13,14]. COPD contributes significantly to morbidity and mortality in Aotearoa NZ, with Māori experiencing higher incidence and mortality rates [14]. In 2015, COPD-related mortality among Māori aged ≥45 years was more than twice that of Europeans [15,16].

High quality international studies have shown that lung cancer screening (LCS) is effective at reducing mortality from lung cancer by more than 20% [17,18]. Two large trials, the US National Lung Screening Trial (NLST [17,18] and the Netherlands / Belgium (NELSON) trial [17,19] found reduced mortality when high-risk people were screened for lung cancer using a low dose CT scan (LDCT). A recent Cochrane Review of 11 trials (N = 94,445) [20], including NLST and NELSON, confirmed these findings as did an earlier meta-analysis of nine trials [21] and a further review [22]. The International Lung Screening Trial (ILST) is underway in multiple centers worldwide including Australia, Canada, Hong Kong and Spain [23,24] and a further NELSON implementation trial (N = 26,000), the 4-In-the-Lung-Run consortium based in nine centers in six European countries, intends to evaluate implementation factors [25]. These trials will provide further information regarding the efficiency of screening and improving the balance of harms and benefits associated with screening. Current best practice recommendations for optimizing LCS program parameters include using follow-up CTs with volumetric assessment, incorporating lung health checks such as COPD assessment as well as integrated smoking cessation [26]. There is consensus on many aspects of the screening pathway, with some well-articulated knowledge gaps, and strong recommendations to ensure assessment of local context and policy requirements [27,28].

### Indigenous health equity and implementation science

Although there are acknowledged socioeconomic and geographic inequities related to LCS, and some more recent commentary on disparities related to program inclusion criteria [28–32], commentary has been largely silent on the clear research gap about Indigenous health equity in lung cancer screening, and, until recently, in consideration of equity in implementation science more broadly [33,34]. This is important in relation to Indigenous rights to health, as outlined in the United Nations Declaration on the Rights of Indigenous People (UNDRIP) and Aotearoa NZ’s Treaty of Waitangi [35,36], to address the significant lung cancer inequities and to eliminate the contribution that lung cancer makes to the lower life expectancy of the Māori population [5,14,37]. This study approach is grounded in Te Ao Māori (Māori worldview), Indigenous rights and Māori health equity at every step of planning, process, implementation and outcomes, including program parameters and the setting of research questions. Our incorporation of the Te Reo Māori (Māori language) name for the study (Te Oranga Pūkahukahu), and key aspects of Māori leadership and governance, Māori workforce (including Māori specific-roles), Māori data sovereignty, iwi (tribal) and kaumātua (elder) support and Māori consumer participation and co-design, are covered in the CONSIDER statement checklist [38] (S1 Appendix).

No LCS program currently exists in Aotearoa NZ. Our study will provide policy-relevant evidence for the implementation of LCS in Aotearoa NZ. LCS has great potential to improve Māori LC outcomes and reduce inequities (particularly for Māori women) [37], providing that we: do not underestimate Māori risk and can identify the high-risk screening population; achieve equitable uptake; deliver equitable and effective treatment; and ensure that harms are not greater for Māori.

The current study was informed by our prior survey results indicating equipoise between proposed invitation approaches based in primary care or delivered by a program ‘hub’ (contact center) [39]. This study therefore compares these two primary invitation methods. By identifying the most successful primary invitation method for Māori, the basis of a successful screening program can be developed. Our study also takes up the opportunity for screening programs to provide co-benefit interventions, by nesting a sub-study on COPD within the overall RCT. This is particularly relevant given that LC and COPD share risk factors that contribute to morbidity and mortality, and that COPD is a risk factor for LC. Alongside the central research question, the study uses the RE-AIM framework to evaluate specific implementation factors.

## Methods

### Objectives

The objectives of the trial relate to both the cluster and individual participant level. The primary objective (cluster level) is to determine the effectiveness of two LCS invitation strategies (invitation and risk assessment via primary care/general practice (clinics) versus invitation and risk assessment via a centralized contact ‘hub’) for increasing screening uptake by Māori. Secondary objectives are to: 1) describe key LCS outcomes required to inform a potential national LCS program in Aotearoa NZ (cluster level); 2) to evaluate implementation factors to optimize such a program (using the RE-AIM framework) (cluster/participant level); and 3) to determine how COPD assessment within a LCS setting impacts the management of patients with COPD (participant level).

### Hypothesis

We hypothesize that the invitation to LCS conducted in primary care will result in higher levels of participation by Māori than a central hub, due to the trusted relationships between patients and their primary care providers.

### Design

**Fig 1:**
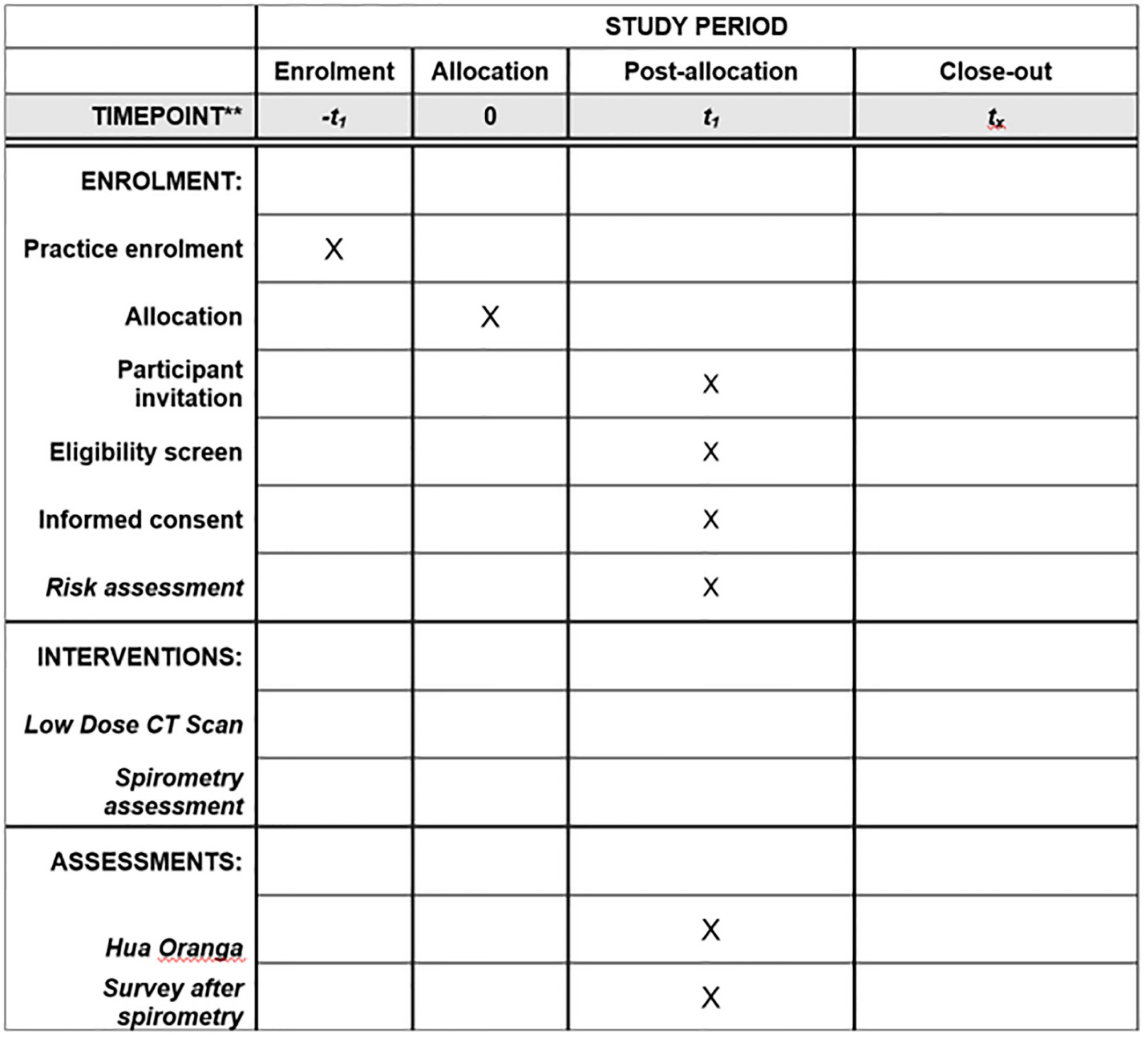
Outline of study components as per the SPIRIT checklist.

**Fig 2:**
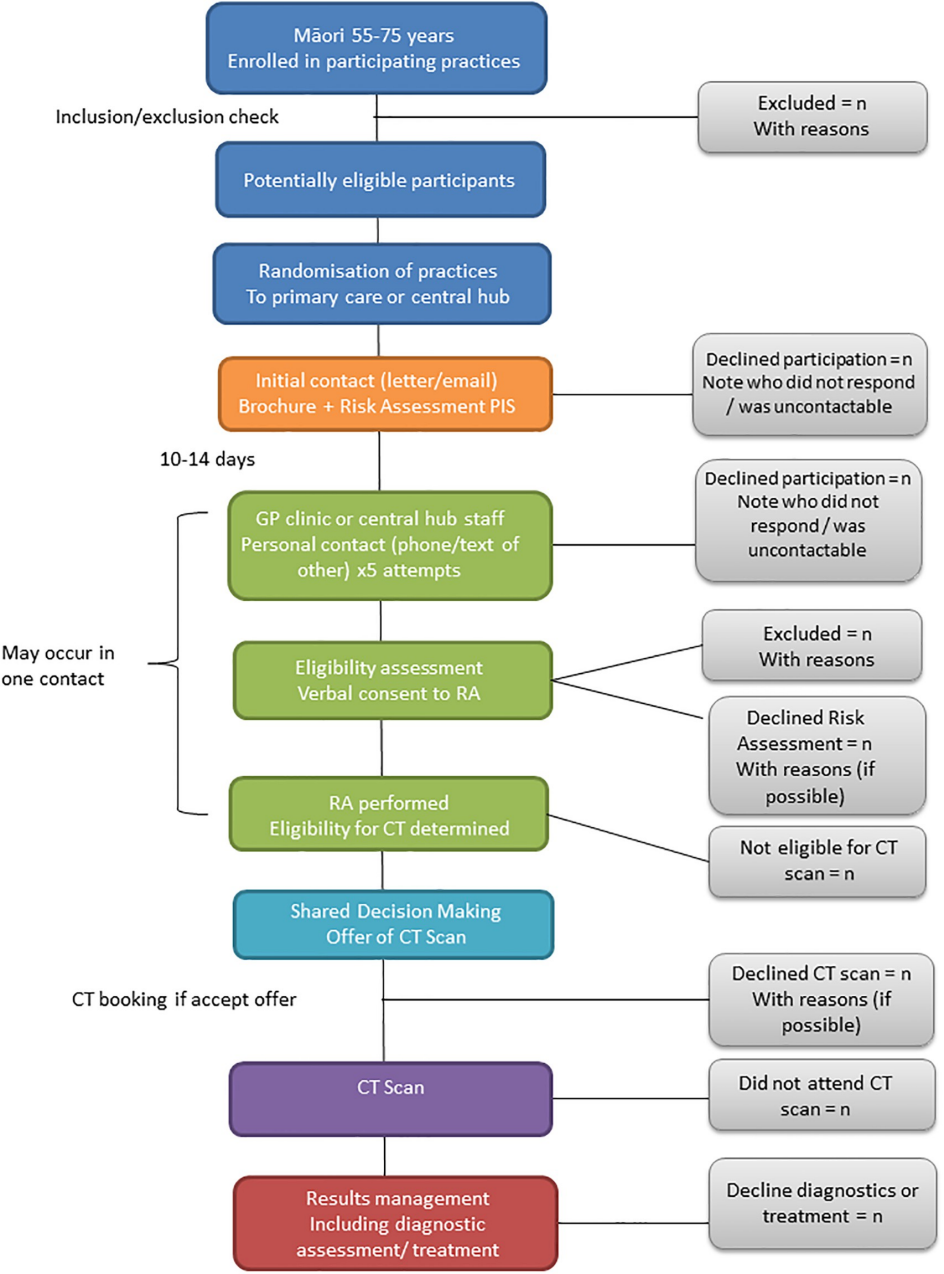
Overall schematic of study process.

**Fig 3:**
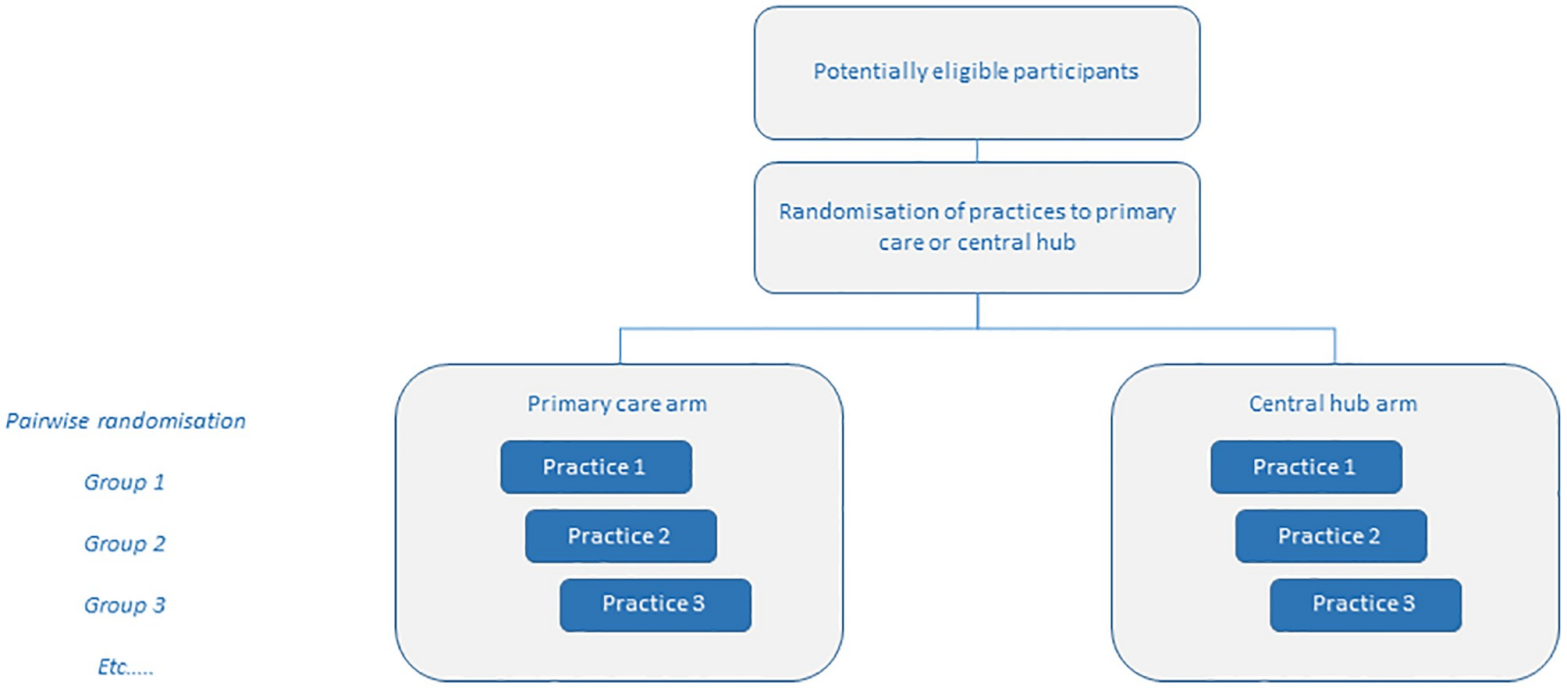
Pairwise practice randomization schematic (the groups will extend until we reach the required number of practices—Up to a maximum of 48 (excluding pilot practices)).

This study is a pragmatic, community-based cluster randomized controlled trial (RCT) conducted within the greater/metropolitan Auckland region of Aotearoa NZ. The SPIRIT guidelines [40] have been followed and Fig 1 outlines when each study component occurs. A nested cohort sub-study on COPD will also be undertaken. An overall study schematic is outlined in Fig 2. Recruited practices will be randomised in a pairwise fashion, with one of each pair of practices being ransomised to each of the study arms (Fig 3).

### Eligibility criteria

Eligible clinics will be those who are physically located in specified large urban geographic districts in Aotearoa NZ’s largest city, Auckland. Clinics will be excluded if they are physically located outside of these districts.

Eligible participants will be Māori (self-defined) aged 55–74 years who are enrolled in participating clinics in the study region and are ‘ever smokers’. Ever smokers includes both current and ex-smokers, classified in the primary care data extract (coded in the practice management system) as anyone who is not a never-smoker/non-smoker. In Aotearoa NZ, ethnicity is self-reported with multiple ethnicity recording possible. Ethnicity is collected in clinics according to the Ethnicity Data Standards [41], with all ethnic groups extracted and reported using prioritization to a single ethnic group (Māori>Pacific>Asian>other) where multiple ethnicities are recorded. The age range for LCS internationally is a contentious issue and the range for this trial generally aligns with main international trials [18,23,24]. Participants will be excluded if they have: a previous diagnosis of lung cancer; another cancer that is non-curative; a presence of any symptoms suspicious for lung cancer; received chemotherapy or cytotoxic drugs within the last six months (except for methotrexate treatment for rheumatoid arthritis); and/or have had a chest CT within the last two years. Participants will also be excluded if they are pregnant and/or are unwilling or unable to provide informed consent.

### Recruitment

#### Primary care recruitment

Clinics are overseen by Primary Health Organizations (PHOs), who will compile lists of potentially eligible Māori (as outlined in Fig 1). Each participating clinic will review the list to confirm participant eligibility and, on behalf of clinics, the study will send potentially eligible people an initial letter or email outlining the study, noting their doctor’s endorsement and inviting the person to participate (it reads as if it is sent by the clinic).

#### Central hub recruitment

Clinics will provide the finalized list of eligible people, along with contact details and other demographic data relevant to the study, to the study team. The study team will send potentially eligible people an initial letter or email outlining the study and inviting the person to participate.

### Informed consent

Ten to 14 days after invitations are sent, the clinic (primary care arm) or study team (central hub arm) will follow up with people by phone or text to further explain the study and to undertake the consent process (if the person wishes to participate). As risk assessment is telehealth/phone based, verbal consent for risk assessment will be obtained by asking the participant if they wish to proceed with a risk assessment. This consent, if given, will be documented in the electronic trial record at the time. The risk assessment will not proceed unless the participant gives consent. If the person’s level of risk is higher than the threshold for eligibility for a LDCT scan, a shared decision making process will be undertaken to support their decision making about having a LDCT scan. If they chose to proceed with the LDCT scan, written consent will be obtained prior to the scan being undertaken. Written consent to access relevant respiratory health data in primary care and hospital records and undergo spirometry in the COPD component will also be obtained. All written consents will be witnessed by the study nurse. The methods of obtaining verbal consent for risk assessment, and written consent for LDCT scan and COPD-related aspects of the study were approved by the NZ Central Health and Disability Ethics Committee (HDEC).

### Randomization and blinding

Participating clinics will be randomized pairwise 1:1 to either the primary care-led or central hub arm. The randomiszation sequence is computer generated at the start of the study using a random number generation formula. For each pair of practices recruited, a sealed envelope containing an instruction that either the first or the second in the pair is to be placed in the primary care-led arm is opened. There are equal numbers of envelopes with each instructions and the envelopes are randomly sorted). Pairwise randomization ensures that there are equal numbers of practices in each group and it means that not all practices need to be recruited from the outset, a distinct advantage when recruiting primary care practices in the current, Covid-constrained environment.

### Interventions

#### Risk assessment

Practice nurses (primary care arm) or the project research nurses (central hub arm) will undertake the risk assessment in-person or via phone or Zoom (Video Communication Inc), according to participant preference. A standardized protocol for completing the risk assessment will be used; staff in both arms of the trial will receive training in this and a procedure manual is provided to everyone undertaking these assessments. Whānau (family) or other support people can participate in the risk assessment process.

The PLCO_M2012_ risk prediction tool [42] will be used to assess a participant’s risk of lung cancer. Participants with a ≥ 2% risk of developing lung cancer within six years will be eligible for a CT scan. This risk threshold has been selected because it is an intermediate point within the range used internationally. The ≥ 2%/6*y* risk threshold was used recently in Canada for a health technology assessment and is currently in use in Ontario Health-Cancer Care Ontario’s *Ontario Lung Screening Program* [43]. We are using an ethnic weighting based on evidence from Tammemägi [44].

People with a ≥30 pack-years smoking history will be eligible for LCS even if their PLCO_M2012_ derived risk is below the 2% threshold. Pack-year is defined as number of packs of cigarettes smoked per day multiplied by the number of years smoked. If a participant stopped smoking for 6 months or more and then restarted smoking again, the time will be subtracted from the total duration of smoking in 0.5-year increments.

#### Radiology

Scans will be undertaken at certified private community radiology providers whose scanners meet the minimum specifications for LDCT scan dose and slice thickness parameters. Participants will be met at the CT scan by a whānau engagement coordinator (a Māori-specific role assisting with providing a positive scan experience and providing support and a warm handover to the research nurses). Participants can bring whānau or other support people with them.

The radiology provider will provide a summary report on the imaging stating that the scan was conducted for research. The scan images will then be sent to the study radiologist based in a secondary or tertiary center.

The study radiologist will complete a standardized proforma report describing the findings, including: lung nodules, structural lung and airways disease and other incidental findings. Where nodules are present, lung nodule volumetry will be performed and recorded in the report. The report will be sent to the participant’s primary care doctor (general practitioner), the study respiratory physician and the study coordinator. Both positive and negative results will be returned to the participant.

A variety of lung nodules may be detected by the CT scan. All identified nodules will be followed up using the PanCan nodule risk management classification system to determine the malignancy risk of a nodule [44,45]. Where required, follow up of participants at risk will be via referral to the local respiratory service who will arrange surveillance scans and other follow-up as required. Malignancy risk is determined by nodule characteristics including site (lobe, juxta-pleural, perifissural), volume, density, presence or absence of spiculation or a benign pattern of calcification, and nodule type (solid (SN), part-solid nodules (PSN) or pure ground glass nodules (pGGN)). Solid nodules with benign features, popcorn calcification, and intrapulmonary lymph nodes will be noted in the report, but do not require surveillance.

The total number of nodules and other findings will be recorded. Where multiple nodules are detected, at least two, including the largest, will be characterized. Low and moderate risk nodules identified on the screening scan will have follow-up scans to assess growth (whereas high risk/suspicious nodules will be referred for diagnosis, as above). Malignancy risk thresholds and the actions associated with each level of risk are described in Table 1.

**Table 1:** PanCan nodule malignancy risk and associated actions for screening scan.

Category	PanCan nodule malignancy risk at screening scan	Action
1	No significant nodules: normal finding, nodule risk < 1.5%	No further action
2	Low risk: nodule risk 1.5% to < 6%	Repeat CT scan at 12 months (follow up scan) No growth at 12m: repeat in further 12m and, if no growth then no further action*. Any interval growth at 12 m: refer for diagnosis
3	Moderate risk: nodule risk 6% to < 30%	Repeat CT scan at 3m (follow up scan) 3m scan result: No growth: repeat scan at 12m intervals until no growth for 2 years and then no further action If interval growth at 3m or any of the subsequent scans: refer for diagnosis Consider referral for diagnosis if nodule risk 10 to < 30%, or other features suggesting benefit from surveillance.
4	High risk: nodule risk ≥ 30%	Refer for diagnosis
5	Suspicious: mass/lesion; mediastinal or hilar lymphadenopathy irrespective of nodule size	Refer for diagnosis

*at the radiologist’s discretion, follow up may be extended for ground glass opacifications, as these are slower growing.

New nodules detected on a follow-up scan will be managed differently to pre-existing nodules as there are significantly different implications for being a lung cancer.

#### COPD assessment

This nested cohort study will determine how COPD assessment within a LCS setting impacts the management of patients with COPD. Participants undergoing a LDCT scan who consent to participate in the COPD study will undergo COPD assessment immediately after their CT scan. The assessment will be undertaken by trained study nurses. The assessment will include the COPD Assessment Test [46,47] and spirometry. Spirometry will be undertaken using a standardized process with the highest value of the best three acceptable blows used for classification of COPD status [48,49]. COPD will be diagnosed by a forced expiratory volume (FEV1) to forced vital capacity (FVC) ratio of less than 70% of the expected result for that participant’s age and sex (FEV1/FVC < 0.70) [48,49]. The severity of obstruction with be classified as mild (FEV1/FVC < 0.7 and FEV1 60–80% of predicted), moderate (FEV1/FVC < 0.7 and FEV1 40–59% of predicted), and severe (FEV1/FVC < 0.7 and FEV1 <40% of predicted).

Participant information about their medications, respiratory-related hospitalizations and recording of COPD diagnosis and previous spirometry results will be extracted from their GP and hospital records and confirmed by the participant. Other information includes COPD-related changes in the T0 LDCT screening scan; COPD status at baseline (known/not known) and, where COPD was previously recorded, assessment of COPD management against COPD guidelines [50,51].

Immediately following the COPD assessment, the nurse will explain results to the participant. A standardized report that includes whether the participant has COPD, its severity and recommendations for COPD management based on NZ guidelines will be sent to the participant’s general practitioner (GP). Four months later, data will be collected from the GP record, to determine whether the GP’s management of COPD has changed from baseline.

### Outcomes of interest

The primary outcomes of the invitation trial are:

#### Cluster level

1) the proportion of eligible population who completed a risk assessment and 2) the proportion of people eligible for a CT scan who completed the CT scan. These proportions will be compared across the two arms of the trial, to determine the answer to our primary research question.

#### Cluster/Participant level

The secondary outcomes provide other information needed to inform the development of a national screening program. The outcomes are described in Table 2 using the RE-AIM implementation science framework [52].

**Table 2:** Implementation science dimensions and outcomes for the invitation trial.

Dimension	Element/level	Outcomes
Reach	Describe target audience & proportion reached (cluster)	Participation proportion for Primary outcome 1—proportion of eligible population who completed risk assessment. Number excluded and reasons. Record contact type responded to (letter, phone, SMS, social).
	Comparison of sample to the target population (cluster)	Representativeness of risk assessment participants and non-participants (by age, gender and smoking history). Representativeness of CT scan participants compared to those eligible for CT scan but who did not consent to one/attend for scan.
Effectiveness	CT scan uptake (cluster)	Participation proportion for Primary outcome 2—proportion of people eligible and who complete CT scan, by invitation arm. Number excluded and reasons.
	Quality of life: Participant experience (participant)	Based on the dimension-based (Hua Oranga) questionnaire centered around four aspects of wellbeing: spiritual, mental, physical, family and including specific questions on anxiety—explored further in the interview [53]. Participant interview including anxiety and appropriateness of SDM, level of information, study materials and value of screening to participant and whānau.
Adoption	Staff (participant)	Number and description of delivery agents (e.g. practice nurse, general practitioner) per practice. Number and description of non-participants. Number of staff attending standardized training session including an implementation manual and guide to the advanced form data entry system embedded in their Practice Management System (PMS). Pre and post training survey of knowledge and confidence, particularly in providing SDM. Survey includes ability to feedback to improve future training. For all above measures, timelines will also be monitored.
	Setting (cluster)	Proportion of primary care practices offered participation who choose to participate. Characteristics of participating and non-participating practices.
	System impacts	Audit against readiness assessment assumptions.
Implementation	Setting—primary care and secondary services (cluster/participant)	Number of claims & cost for completed screen events per practice. Triangulated with completion of the advanced form (checklist tool) for screen event—this tool will capture the key events for fidelity, time to complete the screen, text field for adaptations/issues/fixes to consider. Fidelity triangulated with research nurse observations of risk assessment completion. Ongoing partnership with providers and key stakeholders with iterative feedback including on barriers and adaptations made. Updating our cost effectiveness model with trial data.
Maintenance	Individuals (participant)	Incorporation of Consumer Advisory Group feedback ongoing. Participant and whānau questionnaires including asking whether they would recommend participation to friends & whānau, what more could be done to reach eligible people, recommendations (covers a range of RE-AIM dimensions). Although not within this protocol, we are planning for a (funding dependent) 2^nd^ and 3rd screening round. We propose to contact round 1 participants at 12 months post-intervention to determine willingness to be re-screened. Surveillance scans will be managed by the respiratory team.
	Setting (participant)	Debrief post-trial with key stakeholders including consumer advisory group, primary care and hospital stakeholders. Provider focus groups and/or interviews (covers a range of RE-AIM dimensions).

The outcomes of the COPD sub-study are: the prevalence of COPD among participants who proceed to CT scanning; the characteristics of COPD (GOLD classification, severity); description of how COPD was managed by general practitioners prior to receiving COPD assessment results; change in their management of COPD after receiving the COPD assessment results and management recommendations; participant experience of COPD assessment and participants’ views on the inclusion of COPD assessment in future LCS programs.

### Sample size

#### Invitation trial

Up to 48 clinics will be recruited, with 24 randomized to each study arm (Fig 2).

The sample size for the overall randomized trial is calculated based on the power required to test the hypothesized difference in the number of participants completing LCS following an invitation to the study. Sample size was calculated using the formula cited in Hade et al [54]. S2 Appendix outlines the assumptions made in the sample size calculation. Letters will be sent to 4412 potentially eligible people. Of these, we estimated that 3309 will complete the risk assessment, with 1134 likely to be eligible for LDCT LCS. We estimate that approximately half of those (500–550) will consent to undergo LDCT scan. We expect 400 of these will complete a COPD assessment in conjunction with their LDCT scan. The CT scans conducted in the study will form a baseline screening round (T0).

### Timeframe

We expect that all CT scans and COPD assessments will have taken place by early 2024, with final collation of data in mid-2024.

### Data management and monitoring

A Privacy Impact Assessment has been completed. All participant data will be de-identified. Data will be managed securely in a REDCap (Research Electronic Data Capture) database and also in an electronic study database (’ProCon’) housed at Waitematā district, Te Whatu Ora/Health NZ. The ProCon database is a bespoke IT system which will hold data that is uploaded from practice management systems. It will also hold data on eligibility, inclusion, exclusion and the LCS risk assessment. The data will be uploaded monthly into REDCap.

#### Data sharing and governance

No patient-level data will be shared outside of the research team for this study. A Māori data sovereignty assessment was conducted [55,56], confirming the requirement for Māori data governance. All data in this study will be Māori data and will be governed by the Māori members of the steering group, led by the Principal Investigator, with appropriate protections, data access agreements and management procedures consistent with Te Mana Rauranga (the Māori Data Sovereignty Network) principles [53].

#### Data monitoring

A person independent to the trial will be appointed to monitor the trial conduct. The trial will be monitored after 200 participants have been enrolled, at trial close-out and twice during the trial.

## Discussion

This randomized controlled trial aims to determine the effectiveness of two LCS invitation strategies, primary care or central hub-led, for increasing LCS uptake by Māori. These outcomes will inform any future LCS program in Aotearoa NZ by providing policy-relevant data.

The trial is part of a research program that has intentionally prioritized equity by involving Indigenous Peoples (Māori) in all aspects of research design, analysis and reporting. Our research processes and reporting are aligned with Huria et al’s [38] 17 criteria for reporting health research involving Indigenous Peoples (S1 Appendix). Indigenous rights, the significant and inequitable health impacts of LC for Māori, and the need to ensure that a future LCS program achieves equitable outcomes are key drivers for the research program.

The trial will also provide T0 baseline data of LCS and RE-AIM implementation outcomes to inform equitable program development. Parsimonious design enables the nested assessment of COPD status at baseline, and determination of improvement in COPD management based on tailored reporting to primary care.

The study is world-leading as it is Indigenous-led and designed. As the first clinical study in the Te Oranga Pūkahukahu program, it seeks to answer equity-focused questions on key policy-relevant LCS program parameters.

## Supporting information

S1 Appendix CONSIDER statement: Checklist items. (DOCX)

S2 Appendix Assumptions used in power calculations: Table. (DOCX)

S3 Appendix SPIRIT checklist. (DOC)

S1 File (DOCX)
