# Supplementary material for: Invitation methods for Indigenous New Zealand Māori in lung cancer screening: Protocol for a pragmatic cluster randomized controlled trial
Source: PLoS One. 2023 Aug 1;18(8):e0281420. doi: 10.1371/journal.pone.0281420 (PMC10393155; doi:10.1371/journal.pone.0281420)
Supplement: S1 Appendix — (DOCX) [file pone.0281420.s001.docx]

| **S1 Appendix: CONSIDER Statement: Checklist items**  **‘Te Oranga Pūkahukahu: Lung Health Check’** | |
| --- | --- |
| **Governance** | |
| 1. | Describe partnership agreements between the research institution and Indigenous-governing organization for the research, (e.g., Informal agreements through to MOU (Memorandum of Understanding) or MOA (Memorandum of Agreement)). |
|  | This study is a Māori-led formal partnership between the University of Otago and Te Whatu Ora (Health New Zealand) Waitematā and Auckland districts. There are i relationships with Hei Āhuru Mōwai (the Māori Cancer Leadership Network) and Te Ora (Māori Medical Practitioners Association). Support and approval from Iwi (Ngāti Whātua) and urban Māori (Te Whānau o Waipareira) in the districts the research is occurring was obtained. |
| 2. | Describe accountability and review mechanisms within the partnership agreement that address harm minimization. |
|  | Study governance includes a Māori-led steering group (11 of 13 members are Māori – representing Māori expertise across oncology, public health, primary care, epidemiology, qualitative research and kaupapa Māori methodologies). The steering group had input into the design of the study and have requested regular monitoring of study parameters including potential screening harms (e.g. delay in receipt of results, false positives), as well as risks and issues. The steering group have appointed two advisory bodies – one is a technical reference group (national leaders in primary and secondary care) and the other is a Māori consumer group. This LCS research program was developed with Māori who would potentially be eligible for LCS, through initial focus groups, a survey of beliefs and attitudes, and a Māori consumer advisory group called Te Hā Kotahi. Te Hā Kotahi continue to review study progress and provide advice and consent for new developments in the program. The name Te Hā Kotahi translates as ‘the first breath’, signifying that this advisory group pave the way for future iterations of the LCS program and acknowledging their role as co-designers of the program. The Te Reo Māori program name, developed by Te Hā Kotahi, is ‘Te Oranga Pūkahukahu: Lung Health Check’. The Māori name symbolizes healthy lungs, acknowledging that lung health is a journey and one that is important not only for the individual but for future generations of whānau (family). “Oranga” means “survivor, health, living” and “Pūkahukahu” means “lungs” (as well as the mound at the base of the native Aotearoa NZ Kauri tree that protects the root system of the tree). Other routine review mechanisms include Māori-specific review as part of the ethical and localities review process, and Māori review as part of the research governance approval process with primary care organisations. |
| 3. | Specify how the research partnership agreement includes protection of Indigenous intellectual property and knowledge arising from the research, including financial and intellectual benefits generated (e.g., development of traditional medicines for commercial purposes or supporting the Indigenous community to develop commercialization proposals generated from the research). |
|  | There are no financial, commercialisation or Indigenous intellectual property aspects to this trial. |
| Prioritization | |
| 4. | Explain how the research aims emerged from priorities identified by either Indigenous stakeholders, governing bodies, funders, non-government organization(s), stakeholders, consumers, and empirical evidence. |
|  | Lung cancer is a leading cause of death for Māori and there are serious inequities in incidence and outcomes. It is also a key driver of the life expectancy gap between Māori and non-Māori. Cancer, and specifically lung cancer, has been an identified priority for Māori health and Hei Āhuru Mōwai (the national Māori cancer leadership network) for many years. Early detection and screening to address lung cancer screening outcomes, along with further resource for tobacco control and treatment pathways, are a focus for Te Aho o te Kahu, the Cancer Control Agency, and the two new health agencies in Aotearoa NZ (Te Whatu Ora, Health NZ and Te Aka Whai Ora, the Māori Health Authority). |
| Relationships (Indigenous stakeholders/participants and Research team) | |
| 5. | Specify measures that adhere and honor Indigenous ethical guidelines, processes, and approvals for all relevant Indigenous stakeholders, recognizing that multiple Indigenous partners may be involved, e.g., Indigenous ethics committee approval, regional/national ethics approval processes. |
|  | Initial implementation science (IS) planning used He Pikinga Waiora [1], an Indigenous-developed IS planning tool. Indigenous ethical and data sovereignty principals were incorporated into the discussion, planning and development of the specific research questions. Māori research review was undertaken in Auckland and Waitematā districts of Te Whatu Ora via He Kāmaka Waiora Māori Health Research Services as part of the research locality assessment. Māori leadership, governance and relationships are embedded throughout this project and the whole programme of research, and are consistent with Te Ara Tika [2] ethical guidance for health research. |
| 6. | Report how Indigenous stakeholders were involved in the research processes (i.e., research design, funding, implementation, analysis, dissemination/recruitment). |
|  | As above, this is a Māori-led study with Māori investigators. The steering group provides governance and support from their wide networks; and have been involved in the design and throughout the research process including monitoring, analysis, interpretation and dissemination. Māori workforce (investigators, nursing, data analysis, research assistants, and the Māori-specific role of whānau (family) engagement coordinator) are critical to Māori leadership throughout all levels of the study, but also to ensuring a positive experience for participants throughout. Te Hā Kotahi is a group of Māori and whānau members who are/would be eligible for LCS and have had an essential and ongoing role throughout the programme. |
| 7. | Describe the expertise of the research team in Indigenous health and research. |
|  | The principal investigator (P.I.) of this study is Prof Sue Crengle, who is Māori, a GP and public health physician and a senior Māori health researcher. Dr Rawiri McKree Jansen is Māori, a GP and experienced in Māori health research.Prof Crengle is currently a Board member and Dr McKree Jansen has recently been appointed chief medical officer of Te Aka Whai Ora – The Māori Health Authority. Dr Sandra Hotu is Māori and has expertise in Māori health and respiratory disease. There is a Māori PhD student. The day-to-day study team, including the study research nurses, a Māori engagement coordinator and other staff involved in interacting with Māori participants, are Māori. Other members of the research team have relevant and wide-ranging experience relating to Māori health research and/or other Indigenous research. |
| Methodologies | |
| 8. | Describe the methodological approach of the research including a rationale of methods used and implication for Indigenous stakeholders, e.g., privacy and confidentiality (individual and collective) |
|  | The methodological approach is consistent with the principles of Kaupapa Māori research methodology. Quantitative methodologies are used in the clinical trial and implementation science components of the project. |
| 9. | Describe how the research methodology incorporated consideration of the physical, social, economic and cultural environment of the participants and prospective participants. (e.g., impacts of colonization, racism, and social justice). As well as Indigenous worldviews. |
|  | The initiation of the programme of work with focus groups and surveys with potentially eligible Māori, and the subsequent development of the consumer advisory group Te Hā Kotahi demonstrates best practice in ensuring co-design with Māori who potentially would be impacted by a screening programme. Māori research team members (principal and co-investigators, research assistant, data analyst, nursing and the Māori-specific role of whānau engagement coordinator) are important roles to the success of study – those involved in study design, analysis and interpretation and those frontline staff interacting with whānau- to ensure that participants and whānau are welcomed (manākitanga), respected, and supported throughout the process. Careful consideration was given to the presentation of study materials, both the content in terms of health literacy and the visual resonance and appeal. These were developed by a Māori communication specialist and tested with the Māori consumer advisory group, Te Hā Kotahi. The projects described in this protocol only have Māori participants. |
| Participation | |
| 10. | Specify how individual and collective consent was sought to conduct future analysis on collected samples and data (e.g., additional secondary analyses; third-parties accessing samples (genetic, tissue, blood) for further analyses). |
|  | The study used individual consent, with whānau involvement and discussion encouraged and supported. Consent was sought to re-contact participants with planned follow up interviews, and consent specifically sought for access to medical records including for follow up in both LCS and COPD components. As per the Māori Data Sovereignty assessment, data will not go out of Aotearoa NZ and will not be shared outside of the research group. |
| 11. | Describe how the resource demands (current and future) placed on Indigenous participants and communities involved in the research were identified and agreed upon including any resourcing for participation, knowledge, and expertise. |
|  | Potential participant burden was considered throughout design and is a specific question in the RE-AIM study outcomes, particularly the inclusion of the COPD sub-study. This was discussed explicitly with investigators and the steering group. Koha (reciprocity) in the form of vouchers was included as a key element of study design, including for survey feedback, and transport support is covered by the study to address a key participation barrier. Te Hā Kotahi members are paid for their time and expertise at meetings, and the meetings include knowledge sharing at their request by wider research networks (for example presentations on CT scan technology, logo design and development processes, genetics/biobanking, Te Reo (Māori language) support by local kaumātua (elders)). |
| 12. | Specify how biological tissue and other samples including data were stored, explaining the processes of removal from traditional lands, if done, and of disposal. |
|  | A separate small sub-study is investigating biological samples within Te Oranga Pūkahukahu. No tissue is collected or stored for the studies described in this protocol. |
| Capacity | |
| 13. | Explain how the research supported the development and maintenance of Indigenous research capacity (e.g., specific funding of Indigenous researchers). |
|  | Senior Māori investigators and clinicians alongside Māori research team members are involved in this study. Opportunities for further capacity development, such as Māori public health trainees, are actively sought and supported. |
| 14. | Discuss how the research team undertook professional development opportunities to develop the capacity to partner with Indigenous stakeholders? |
|  | The team is Māori-led, with non-Māori working in partnership as investigators and team members. The position description and interview process for all roles includes a focus on health inequities and drivers, knowledge of Te Ao Māori, and tikanga (the correct way to do things). The team undertake shared professional development opportunities (e.g. external speakers) as well as individual opportunities (conferences, courses) and knowledge sharing amongst team members. Discussions with Indigenous colleagues in other countries is facilitated through research partnerships (e.g. in Australia and Canada) and by the funder Global Alliance for Chronic Diseases (GACD). |
| Analysis and interpretation | |
| 15. | Specify how the research analysis and reporting supported critical inquiry and a strength-based approach that was inclusive of Indigenous values. |
|  | The primary outcome variables are focused on elements of the lung cancer screening program, primary care and hub delivery of LCS and COPD management. Implementation science outcomes describe participants’ experience of LCS and COPD assessment and seek their views on how LCS and COPD assessment could be improved to ensure that Māori experience of (and outcomes from) these in the future will be enhanced. |
| Dissemination | |
| 16. | Describe the dissemination of the research findings to relevant Indigenous governing bodies and peoples. |
|  | The research team will share results with participants as well as with Te Hā Kotahi; the Māori groups and organisations who support this research (Te ORA – Māori Medical Practitioners and other similar NGOs; Ngāti Whātua and Te Whānau o Waipareira; Te Aka Whai Ora – the Māori Health Authority; Māori leadership and staff within Health New Zealand); and will present at specific meetings and conferences with Māori participants. |
| 17. | Discuss the process for knowledge translation and implementation to support Indigenous advancement (e.g., research capacity, policy, investment). |
|  | Prof Crengle (Māori researcher) will lead the process of knowledge translation, including presentations and writing reports and papers. |

**References:**

1. Oetzel J, Scott N, Hudson M, Masters-Awatere B, Rarere M, Foote J, Beaton A, Ehau T. Implementation framework for chronic disease intervention effectiveness in Māori and other indigenous communities. Globalization and health. 2017 Dec;13(1):1-3.
2. Hudson M, Milne M, Reynolds P, Russell K, Smith B. Te Ara Tika: Guidelines for Māori research ethics: A framework for researchers and ethics committee members. Auckland: Health Research Council of New Zealand. 2010. Available at: <https://www.hrc.govt.nz/resources/te-ara-tika-guidelines-maori-research-ethics-0> Accessed 7/11/2022
