# Supplementary material for: Invitation methods for Indigenous New Zealand Māori in lung cancer screening: Protocol for a pragmatic cluster randomized controlled trial
Source: PLoS One. 2023 Aug 1;18(8):e0281420. doi: 10.1371/journal.pone.0281420 (PMC10393155; doi:10.1371/journal.pone.0281420)
Supplement: S2 Appendix — (DOCX) [file pone.0281420.s002.docx]

**S2 Appendix: Assumptions used in power calculations (Invitation study):**

| **Parameter** | **Assumed value** | **Justification** |
| --- | --- | --- |
| Proportion of Māori aged 55-74 who meet eligibility criteria to be invited for screening | 24% | Smoking history data collected in our pilot of AAA screening (28%) [1], reduced by 4% to allow for those excluded on other grounds (terminal illness etc.). |
| Intraclass correlation coefficient* | 0.03 | Approximately the median value of those cited in Hade et al [2]. |
| Average number of eligible patients per practice | 24 | From preliminary work based on practice population registers. Practices with >10 expected eligible patients will be approached to participate. |
| Proportion of Māori in eligible age range recorded as ever smokers | 70% | Preliminary audit data from one of the Auckland PHOs. |
| Proportion of not ever smokers eligible who complete risk assessment in the primary care group | 80% | It is difficult to estimate this parameter as there is no similar experience to base it on. However, it would seem reasonable that it is possible to obtain smoking history on 70% of the eligible practice population in the control group, and that this could be 10% higher in the intervention group where primary care may have a significant advantage. |
| Proportion of not ever smokers eligible who complete risk assessment in central hub group | 70% |  |
| Screening uptake in the central hub group | 40% | Conservative estimate lower than uptake rates routinely achieved for Māori in other screening programs including bowel screening. |
| Screening uptake rate in primary care group | 50% | A finding of 10% higher uptake rate than the control group is both achievable and clinically meaningful. |
| Variance reduction from regression adjustment of individual-level outcomes | 10% | Consistent with variance reduction assumptions used by Hade et al. |
| Variance reduction from regression adjustment of group-level outcomes | 20% | Consistent with variance reduction assumptions used by Hade et al. |
| Statistical power to detect 10% increase in screening uptake | 80% | Standard statistical power selection. |
| Acceptable Type I error rate | 5% | Standard statistical significance selection. |

#### *Although we are randomizing by practice, the units for determining outcome measures are individuals. The intraclass correlation coefficient (ICC) is a measure of how similar individuals within a practice are to each other compared to anyone else in the study. This is important as it affects the sample size calculation substantially. The ICC is not known in advance, so is an assumption based on typical values (gauged from a literature search). Note that while the ICC is normally used for continuous measures, it can also be used for discrete outcomes (as done by Hade et al) and therefore it was appropriate to use here (ie to describe proportions attending LCS).

**Assumptions used in power calculations (COPD sub-study):**

International studies have found high acceptance of spirometry within LCS programs (>90%) [3-4]. We conservatively estimate that 400 (80%) of our LCS participants will consent to COPD assessment. Of these, we assume at least 200 (50%) will meet the criteria for a COPD diagnosis, for whom we can assess changes in baseline management after sending the report to the GP. Some improvement in COPD management over time may be expected following a spirometry test simply due to (normal) ongoing management by their GP or by the Hawthorne effect (i.e. participants modifying their behaviour due to knowledge of being observed) from being included in the study and having a CT and/or spirometry test. We expect this to be seen in about 10% of patients and if the proportion with improvements is significantly greater than 10%, then this might be attributable to the effect of the reporting and management recommendations that we will be providing to the GP. With a sample of 200 the study has 85% power to detect that a measured improvement in management is significantly greater than 10% when the actual proportion is 17% or more (95% significance level).

**References:**

1. Sandiford P, Grey C, Salvetto M, Hill A, Malloy T, Cranefield D, Bramley D. The population prevalence of undetected abdominal aortic aneurysm in New Zealand Māori. Journal of Vascular Surgery. 2020 Apr 1;71(4):1215-21.
2. Hade EM, Murray DM, Pennell ML, Rhoda D, Paskett ED, Champion VL, Crabtree BF, Dietrich A, Dignan MB, Farmer M, Fenton JJ. Intraclass correlation estimates for cancer screening outcomes: estimates and applications in the design of group-randomized cancer screening studies. Journal of the National Cancer Institute Monographs. 2010 Apr 1;2010(40):97-103.
3. De Koning H, Van Der Aalst C, Ten Haaf K, Oudkerk M. PL02. 05 effects of volume CT lung cancer screening: mortality results of the NELSON randomised-controlled population based trial. Journal of Thoracic Oncology. 2018 Oct 1;13(10): S185.
4. National Lung Screening Trial Research Team. Aberle DR, Adams AM, Berg CD, Black WC, Clapp JD, Fagerstrom RM, Gareen IF, Gatsonis C, Marcus PM and Sicks JD: Reduced lung-cancer mortality with low-dose computed tomographic screening. N Engl J Med. 2011; 365:395-409.
