# Supplementary material for: Invitation methods for Indigenous New Zealand Māori in lung cancer screening: Protocol for a pragmatic cluster randomized controlled trial
Source: PLoS One. 2023 Aug 1;18(8):e0281420. doi: 10.1371/journal.pone.0281420 (PMC10393155; doi:10.1371/journal.pone.0281420)
Supplement: S1 File — (DOCX) [file pone.0281420.s004.docx]

A study to assess the effectiveness of a primary care vs central hub invitation to lung cancer screening for Māori participants aged 55-74 in Auckland and Waitematā District Health Boards

| **Research reference number:** | RM14751 | | | |
| --- | --- | --- | --- | --- |
| **Lay title:** | Optimising lung cancer screening for Māori: A study of comparative invitation processes | | | |
| **Trial registration number & date:** | ACTRN12621001309875p 27^th^ September 2021 | | | |
| **Ethics reference:** | 21/CEN/174 | | | |
| **Protocol version number:** | V3.5 March 2022 | | | |
| **Sponsor:** | Dr Debbie Holdsworth, Director Funding  Waitematā District Health Board (DHB) and Auckland DHB | | | |
| **Principal Investigator:** | Professor Sue Crengle^1^ | sue.crengle@otago.ac.nz | 021 832 346 |  |
| **Co-Investigators** | | | | |
| **DHB Project Lead:** | Dr Karen Bartholomew^2^ | Karen.Bartholomew@waitematadhb.govt.nz | 021 211 5629 |  |
| **Programme manager** | Dr Kate Parker^2^ | Kate.Parker2@waitematadhb.govt.nz | 021 678 907 |  |
| **Oncologist** | Professor Mark McKeage^4^ | MarkM@adhb.govt.nz | 021 859 588 |  |
| **Data analysis and statistics** | Dr Peter Sandiford^3^ | Peter.Sandiford@waitematadhb.govt.nz |  |  |
| **Respiratory physician** | Dr Chris Lewis^5^ | CLewis@adhb.govt.nz | 021 667 569 |  |
| **Radiologist** | Dr David Milne^5^ | DMilne@adhb.govt.nz | 021 687 507 |  |
| **Primary care physician** | Dr Rawiri McKree Jansen^6^ | Rawirimj@nhc.maori.nz | 021 729 474 |  |
| **Risk prediction modelling expertise** | Professor Martin Tammemagi^7^ | ctammemagi@brocku.ca |  |  |
| **Implementation science expertise** | Dr Nicole Rankin^8^ | nicole.rankin@sydney.edu.au |  |  |
| **LCS expert advisor** | Professor Kwun Fong^9^ | Kwun.Fong@health.qld.gov.au |  |  |
| **LCS expert advisor** | Dr Henry Marshall^10^ | Henry.Marshall@health.qld.gov.au |  |  |
| **Implementation in primary care expertise** | Dr David Jansen^5^ | Rawirimj@nhc.maori.nz | 021 729 474 |  |
| **Co-Investigators for Biomarker study** | | | | |
| **Biomarker expertise** | Prof Cristin Print^4^  Dr Cherie Blenkiron^4^ | [C.Print@auckland.ac.nz](mailto:C.Print@auckland.ac.nz)  [C.Blenkiron@auckland.ac.nz](mailto:C.Blenkiron@auckland.ac.nz) |  |  |
| **Co-Investigators for COPD study** | | | | |
| **Respiratory physician COPD** | Dr Sandra Hotu^5^ | [SHotu@adhb.govt.nz](mailto:SHotu@adhb.govt.nz) |  |  |
| **COPD expertise** | Assoc Prof Rob Young^5^ | [RobertY@adhb.govt.nz](mailto:RobertY@adhb.govt.nz) | 027 290 2266 |  |
| **Spirometry, research nurse oversight** | Raewyn Scott^5^ | [RHopkins@adhb.govt.nz](mailto:RHopkins@adhb.govt.nz) |  |  |
| **Māori qualitative researcher** | Dr Rachael Brown^6^ | [RachelB@nhc.maori.nz](mailto:RachelB@nhc.maori.nz) |  |  |
| **Co-Investigators for Workplace exposure study** | | | | |
| **Lead researcher** | Dr James McLeod^17^ | [james.mcleod@worksafe.govt.nz](mailto:james.mcleod@worksafe.govt.nz) | 021 846 567 |  |
| **Study Team** | | | | |
| **Research Assistant** | Billie Davis^2^ | [Billie.Davis@waitematadhb.govt.nz](mailto:Billie.Davis@waitematadhb.govt.nz) | 021 176 7818 |  |
| **Research Assistant** | Tayla Schaapveld^2^ | [Tayla.Schaapveld@waitematadhb.govt.nz](mailto:Tayla.Schaapveld@waitematadhb.govt.nz) | 027 805 6404 |  |
| **Study Nurse** | Grace Steel^2^ | [Penelope.Steel@waitematadhb.govt.nz](mailto:Penelope.Steel@waitematadhb.govt.nz) | 022 353 4310 |  |
| **Nurse Research Fellow** | Anne Fraser^2^ | [Anne.Fraser@waitematadhb.govt.nz](mailto:Anne.Fraser@waitematadhb.govt.nz) | 021 840 292 |  |
| **Engagement Co-ordinator** | Donna Enoka^2^ | [Donna.Enoka@waitematadhb.govt.nz](mailto:Donna.Enoka@waitematadhb.govt.nz) | 021 949 685 |  |
| **Assistant Research Fellow** | Sarah Colhoun^1^ | [sarah.colhoun@otago.ac.nz](mailto:sarah.colhoun@otago.ac.nz) | 021 030 2666 |  |
| **PhD Student** | TBD |  |  |  |

| **Study Steering Group** | | **Study Technical Advisory Group** | |
| --- | --- | --- | --- |
| Prof Sue Crengle^1^  Ms Aroha Haggie^11^  Dr Rawiri Jansen^6^  Dr Chris Lewis^5^  Ms Sharon McCook^11^  Ms Shelley Campbell^12^ | Dr George Laking^5^  Dr Dale Bramley^3^  Dr Rob McNeill^4^  Dr Nina Scott^13^  Dr Jacquie Kidd^14^  Dr Melissa McLeod^1^ | Professor Marg Wilsher^5^  Professor Mark McKeage^4^  Professor Cristin Print^4^  Dr Claire Hardie^15^  Dr James Entwisle^16^ | Dr Chris Lewis^5^  Dr David Milne^5^  Dr Laird Cameron^5^  Dr Paul Dawkins^11^  Dr Rawiri Jansen^6^ |

1. University of Otago
2. Planning Funding and Outcomes, Waitematā DHB and Auckland DHB
3. Waitematā District Health Board, Shea Terrace, Takapuna, Auckland, New Zealand.
4. University of Auckland
5. Auckland District Health Board
6. National Hauora Coalition
7. Brock University, Ontario
8. University of Sydney
9. Prince Charles Hospital, Queensland
10. University of Queensland
11. Counties Manukau District Health Board
12. Cancer Society NZ
13. University of Waikato
14. Auckland University of Technology
15. MidCentral District Health Board
16. Capital and Coast District Health Board
17. Worksafe

This trial is supported via funding from HRC and the Global Alliance for Chronic Diseases

Contents

[Study oversight 5](#_Toc83021308)

[Synopsis 6](#_Toc83021309)

[Māori advancement statement 8](#_Toc83021310)

[Introduction 8](#_Toc83021311)

[Background 9](#_Toc83021312)

[COPD 11](#_Toc83021313)

[Research Programme 13](#_Toc83021314)

[Aims and objectives 13](#_Toc83021315)

[Pilot phase 14](#_Toc83021316)

[Full trial 14](#_Toc83021317)

[Study Design 15](#_Toc83021318)

[Study setting/Location 15](#_Toc83021319)

[Study Population 16](#_Toc83021320)

[Eligibility Criteria 16](#_Toc83021321)

[Deferrals 17](#_Toc83021322)

[Study Procedures/Methods 17](#_Toc83021323)

[Recruitment of participants 17](#_Toc83021324)

[CT scan 20](#_Toc83021325)

[COPD assessment 20](#_Toc83021326)

[Nodule assessment, classification and management 21](#_Toc83021327)

[CT scan result management and follow up 23](#_Toc83021328)

[Results of COPD assessment 24](#_Toc83021329)

[Secondary research questions 25](#_Toc83021330)

[Smoking cessation advice 28](#_Toc83021331)

[Statistical Consideration and Data Analysis 28](#_Toc83021332)

[Sample size and statistical analysis 28](#_Toc83021333)

[Data management 29](#_Toc83021334)

[Data sharing and governance 29](#_Toc83021335)

[Data and safety monitoring 30](#_Toc83021336)

[Ethical considerations 30](#_Toc83021337)

[Informed consent 30](#_Toc83021338)

[Other ethical issues 30](#_Toc83021339)

[Adverse events and risks 30](#_Toc83021340)

[Outcomes and Significance 32](#_Toc83021341)

[Primary outcome 32](#_Toc83021342)

[Secondary outcomes 32](#_Toc83021343)

[References 33](#_Toc83021344)

[Appendices 36](#_Toc83021345)

## Study oversight

The project is Māori-led by the academic Principal Investigator Professor Sue Crengle (Waitaha, Kāti Māmoe, Kāi Tahu), University of Otago.

A study Steering Group, established in early 2019, is also Māori led and includes DHB Funding and Planning, He Ahuru Mowai, Māori doctors (GPs and oncology), respiratory clinicians and a qualitative researcher; nine of the eleven members are Māori. The group provides us with advice over all aspects of our lung cancer screening programme.

The Steering Group is supported by a Technical Advisory Group, which includes respiratory, oncology, cancer genomics, molecular medicine and radiology specialists and provides technical advice for the lung cancer screening project, a Primary Care Advisory Group and by Te Hā Kotahi, our Consumer Advisory Group, which provides oversight of the research programme (including this study) from a participant and whānau perspective, and ensures our approach is whānau-centred throughout. (Figure 1).


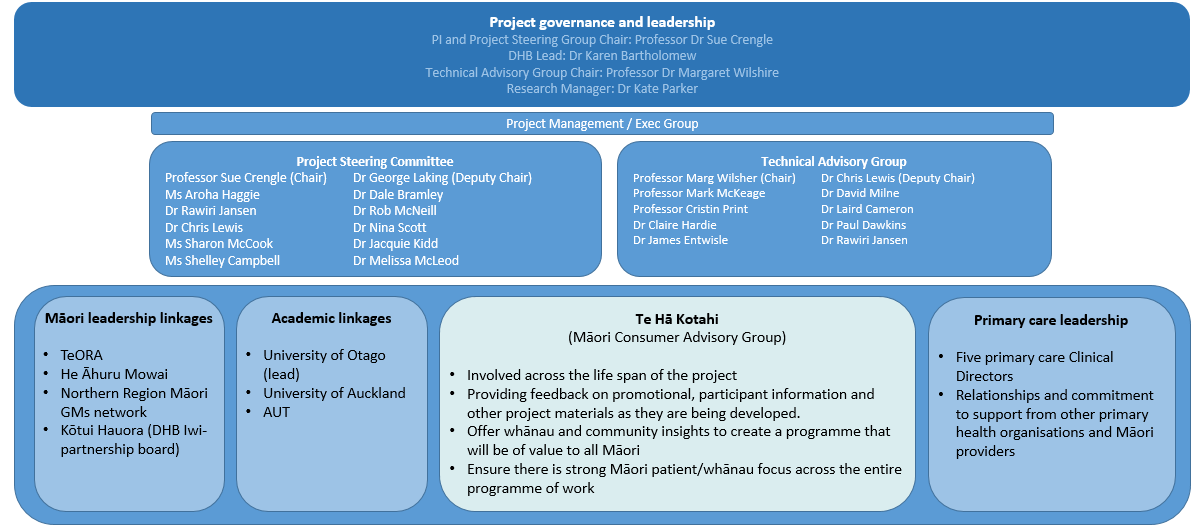


Figure 1: Project structure

Involving whānau from the beginning of the journey is a foundational approach for the research programme and this is facilitated through Te Hā Kotahi, whose members include potentially eligible people and their whānau. The group meets regularly, supported by DHB kaumatua, and has contributed significantly to the framing of the research programme, research questions, participant materials, logo and design and to the name of the programme.

The programme name, Te Oranga Pūkahukahu: Lung Health Check, was proposed by Te Hā Kotahi and has recently been approved by GM Tikanga Dame Naida Glavish. The meaning of the name Te Oranga Pūkahukahu or the Lung Screening Programme is taken from the Māori words “oranga” meaning survivor, food, livelihood, welfare, health, living, and “pūkahukahu” which can refer both to lungs, and to the mound of moss found at the base of the Kauri tree that protects the root system. The overall sentiment of the name symbolises healthy lungs or the desire to have healthy lungs and conveys that the kaupapa is about having healthy lungs and the vehicle to help get there is through lung screening.

## Synopsis

| Study design | Community cluster Randomised Controlled Trial (RCT)  Two lung cancer screening invitation pathways will be tested end to end (invitation, risk assessment, shared decision making and CT scan). Groups will be cluster randomized (at general practice level) 1:1 between central hub and primary hub invitation approaches.  The trial will also include a nested cohort study on Chronic Obstructive Pulmonary Disease (COPD). Participants who are eligible for a CT scan will also be invited for a COPD assessment. |
| --- | --- |
| Timeframe | 3 years from August 2021 to July 2024 |
| Sample population | Māori men and women aged 55 to 74 years, who are not ‘never smokers’ |
| Inclusions | Includes recorded or self-identified as Māori in the primary care enrolment register, aged between 55 to 74, enrolled in participating general practices, able to provide informed consent and able to agree to participate.  Those participants who meet the eligibility criteria to undergo LCS, and who make an informed decision to undergo LCS, will also be invited to participate in the COPD assessment. |
| Exclusions | Never smokers, those with clinical symptoms suspicious for lung cancer, previous diagnosis of lung cancer, have received chemotherapy or cytotoxic drugs within the last six months, and pregnancy; unable to provide informed consent due to cognitive problems; unwilling to provide consent.  Additionally, those unwilling or unable (due to a comorbid disease) to undergo spirometry testing will be excluded from COPD assessment. |
| Expected sample size | Our sample size estimate anticipates that 4412 participants will be invited to undergo risk assessment, with 3309 participants being expected to complete this assessment. Of these, we expect that 1134 participants will be eligible for low dose CT scanning, and that 500-550 participants will consent to undergo LDCT scan for lung cancer screening. After the first 50 participants have been scanned, we will pause the study assess the pathway and methods, to ensure they are optimal before expanding the study to the next 500 scans which will test the invitation methods. Should the proportions of participants agreeing to risk assessment and / or scan differ from our predictions, these numbers will be adjusted accordingly to allow us to reach the 500-550 scan target.  **COPD assessment:**  We conservatively estimate that 400 (80%) of our LCS participants will consent to COPD assessment. Of these, we assume at least 200 (50%) will meet the criteria for a COPD diagnosis, for whom we can assess changes in baseline management after sending the report to the GP. With a sample of 200 the study has 85% power to detect that a measured improvement in management is significantly greater than 10% when the actual proportion is 17% or more (95% significance level). |
| Recruitment | Letter or email sent to potentially eligible participants in both trial arms, with phone or text follow up.  Opportunistic recruitment in primary care arm if people attend the practice for another reason.  All participants will be assessed for their risk of lung cancer and those who proceed to a CT scan will also be invited to take part in a COPD assessment. |
| Aim | This study sets out to determine the effectiveness for Māori who are not “never smokers” of two lung cancer screening invitation strategies; primary care vs central hub.  In doing so, we will describe key outcomes that are required to inform a potential national lung cancer screening programme in NZ, and will evaluate contextual implementation factors including the role of blood-based biomarkers alongside CT scanning in order to optimise a future programme.  The trial will also test the assumptions and planned study processes relevant to the implementation of lung cancer screening in Aotearoa NZ.  We also aim to assess those who participate in lung cancer screening for the prevalence of COPD. We will then follow up those diagnosed with COPD to determine how GPs manage COPD. Participants’ views will inform whether COPD assessment should be included in future lung cancer screening. |
| Objectives | 1. Determine the effectiveness of two invitation strategies for lung cancer screening; primary care vs central hub 2. Describe key lung cancer screening outcomes that are required to inform a potential national programme in NZ, including:    - Cancer detection rates    - Number and proportion of cancers detected by stage and grade    - Proportion of cancers detected that are surgically operable    - Scan positivity    - Types and rates of incidental findings. 3. Evaluate contextual implementation factors in order to optimise a future NZ screening programme. Including the confirmation of key assumptions such as:  - What % of not ‘never smoker’ Māori aged 55-74 in a general practice are eligible for lung cancer risk assessment - What % of people who are eligible for risk assessment agree to undergo risk assessment - What % of those undergoing risk assessment are eligible for lung cancer screening - What % of those eligible for lung cancer screening decide to proceed with screening - What % of those who decide to proceed with screening attend their appointment for screening - Usability of the risk assessment tool - Ability to implement blood biomarker analysis - Data collection and IT systems - Invitation materials - Consent processes - Results management   - Reporting in private community radiology clinic   - Standardised screen read and reporting   - Use of volumetric analysis   - Statistical integration of LDCT information with blood biomarker information   - Interpretation of results by providers and experience of providing results to participants and whānau   - Interface between primary and hospital care for positive screen management - Participant and whānau experience  1. Determine how COPD assessment within a lung cancer screening setting impacts the management of patients with COPD, including:  - Describing the prevalence and characteristics of COPD in those who undergo LDCT in this trial - Describing the primary care management of COPD - Describing any change in primary care COPD classification, recording of spirometry results and management following management recommendations to the participant’s GP - Describing the participant’s experience of COPD assessment and their views on whether it should be included in future lung cancer screening. |
| Test devices / procedures | Low-dose computed tomography  Spirometer  Blood sample biomarker analysis |

## Māori advancement statement

The core focus of this study and of our whole research programme is Māori Health equity. All aspects of the trial design and our approach to each element has equity as the central focus. All data in this study will be Māori data, and will be governed by the Māori members of the steering group, led by the Principal Investigator with appropriate protections, data access agreements and management procedures consistent with Te Mana Rauranga principles.

## Introduction

In Aotearoa NZ lung cancer is a common cause of cancer deaths for Māori and non-Māori. There are significant ethnic inequities in incidence and mortality, particularly for Māori women, with lung cancer being the single largest contributor to the difference in life expectancy between Māori and non-Māori. This cancer burden will persist even if all smokers stopped today, due to the long lag time for smoking attributable lung cancer. Urgent action on lung cancer is required.

Additionally, Chronic Obstructive Pulmonary Disease (COPD) contributes significantly to morbidity and mortality in Aotearoa NZ. Risk factors for COPD include tobacco smoking – this is associated with most COPD cases (85%). COPD increases the risk of lung cancer. Both internationally and in Aotearoa NZ, COPD is the 4^th^ leading cause of death.^1,2^ It is a progressive disease and those who suffer from it experience persistent respiratory symptoms, including shortness of breath, wheezing and coughing as well as many other disruptions to their daily life. It is both preventable and treatable.^1,3^

In 2017, lung cancer was the 4^th^ most common cancer^4^ and the leading cause of cancer death in New Zealand (NZ)^5^. However, for Māori, it is the most common cancer, the leading cause of death for Māori women, and is second to cardiovascular disease for Māori men^6^. Māori women’s rates of lung cancer are over four times those of non-Māori, and in Māori men the rate is nearly three times higher than in non-Māori. Māori also develop lung cancer around six years earlier than non-Māori, and at lower smoking exposures. Māori have approximately 30% higher death rates when diagnosed with cancer, due to a range of factors including comorbidities, later diagnosis, and worse experiences in the healthcare system^7^.

Overall, survival from lung cancer is poor (12% 5-year survival) largely due to late stage at diagnosis. Early stage (stage 1A) lung cancer has a >70% 5-year survival. Earlier detection of asymptomatic lung cancer through lung cancer screening using low dose computerized tomography (LDCT) scans shifts the stage at diagnosis and reduces mortality.

A key issue in the NZ context is developing a screening strategy that will ensure benefit for Māori. Screening programmes for other diseases have typically resulted in inequitable access (participation/coverage) and often persisting or even widening of ethic-inequities in outcomes. Although a range of strategies have been deployed by the National Screening Unit (NSU), no programme has been designed with the intention of ensuring equity and there is ongoing debate as to whether Māori are better served by screening programmes delivered through primary care services or by centralised programmes run by the NSU, Ministry of Health.

Identifying effective strategies to ensure Māori participation in lung cancer screening is imperative. If Māori participation is not at least equal to non-Māori participation, existing serious inequities in mortality will be increased. Systemic changes are required to prevent further ethnic inequities in lung cancer, including in the diagnostic and treatment pathways and in smoking cessation^17^. Achieving equitable cancer care for Māori is a significant health priority, recognised in the New Zealand Cancer Action Plan 2019-2029^8^.

This protocol outlines the first in a series of studies under the broader Lung Cancer Screening Research Programme Te Oranga Pūkahukahu, a collaboration between the University of Otago and Waitematā and Auckland DHBs. This study sets out to determine the effectiveness of two LCS invitation strategies; primary care vs central hub, in recruiting Māori participants to LCS. In addition, the study will also test some of the key assumptions made in planning this first study of LCS in Aotearoa NZ, and will also test key study processes. This will be the first time an organised programme for Lung Cancer Screening has been undertaken in Aotearoa NZ.

## Background

The effectiveness of Lung Cancer Screening has been established. High quality international evidence has shown that screening asymptomatic people at high-risk of lung cancer (LC) with a low dose CT scan (LDCT) is effective at reducing lung cancer mortality^9,10^.

Early detection through LDCT screening of high-risk asymptomatic people has been demonstrated to reduce LC mortality by more than 20%. This has been shown in two large trials, the US National Lung Screening Trial (NLST)^10^, and the Netherlands / Belgium (NELSON) trial^9^. A meta-analysis of these and seven other smaller trials concluded that LDCT screening is associated with a significant reduction in LC mortality^11^.

The NELSON trial used updated LCS pathways that resulted in more efficient screening procedures and far fewer harms. Demonstration sites and screening programmes have been established in a range of countries and there is substantive accumulated experience in the improved risk/benefit profile with risk prediction models, the use of follow up CTs with volumetric assessment rather than invasive diagnostics, incorporation of wider co-benefits such as lung health checks for co-morbidities, such as COPD, and smoking cessation. The NLST has informed a set of national recommendations to implement screening, with guidelines recently updated to extend the eligible age range and reduced smoking history requirements.

A recent European Position Statement outlined the core features where there is consensus and where there are unanswered questions, and this included strong advice to ensure that recommendations are tested and adapted to local contexts.

Of note, none of the current international screening programmes are equity focused or include sufficient representation of Indigenous people, despite the burden of disease in these communities.

There are well recognised knowledge gaps, particularly in relation to equitable lung cancer screening (LCS) access and outcomes, that must be addressed prior to any funding or implementation decisions. Our Māori-led LCS programme team has completed foundational work and is initiating a series of studies of LDCT screening. Our research programme is focused on designing-in equity to the LCS pathway, to ensure that any future national programme will benefit Māori. We have selected LCS because it is an intervention that has great potential to improve Māori LC outcomes and reduce inequities provided that: we accurately identify our screening population, equitable uptake can be achieved, harms are not greater for Māori and there is equitable delivery of effective treatment.

A criticism of LDCT screening is the numbers of false positive results these screens can generate, causing concern to some whānau and using health service resources^52^. Blood plasma biomarkers, especially circulating tumour (ct)DNA have been proposed to increase test specificity and reduce the incidence of these false positive results^53^. Advances on blood plasma ctDNA analysis have provided iterative improvements in sensitivity and specificity for lung cancer detection by LDCT screening, including blood DNA fragmentation profiles^54^ and ctDNA methylation analysis^55^. Other blood biomarkers such as CarcinoEmbryonic Antigen (CEA)^56^, Cancer Antigen-125 (CA125)^57^ and various autoantibodies^58^ have also been used to enhance LDCT screen sensitivity for lung cancer.

Key policy and implementation questions for Aotearoa NZ include: target population (selection of high-risk individuals); ensuring equitable access to LCS for those groups at the highest risk^12,13,14^; invitation approaches; ensuring that the balance of benefits and harms is favourable particularly for Māori who may have higher prevalence of lung findings; management of screen detected abnormalities (nodules and incidental findings); cost-effectiveness using NZ data from LC screening trials; how to best incorporate smoking cessation; and understanding people’s experience of risk assessment and screening, and what support might be required through diagnosis and treatment.

There are two key strategies for improving stage at diagnosis: earlier diagnosis of symptomatic lung cancer and detection of asymptomatic lung cancer. Earlier diagnosis of symptomatic lung cancer has been demonstrated to produce a small stage shift^15^, but earlier detection of asymptomatic lung cancer is associated with greater stage shift with demonstrated reduction in LC mortality.

The Prostate Lung Colorectal and Ovarian (PLCO_m2012_) model is a lung cancer risk prediction model which has been used and validated by multiple lung cancer screening projects internationally^16^. The PLCO_m2012_ is a logistic regression model based on cancer incidence (over 6 years) that occurred in the Prostate, Lung, Colorectal and Ovarian Cancer Screening Trial (PLCO). This model has higher sensitivity, specificity and positive predictive value when compared to the NLST risk prediction criteria – another internationally recognised criterion which defines a person as of “high risk” when they have a smoking history of ≥30 pack years, have smoked within the past 15 years, and are aged between 55–74. The PLCOm2012 model was developed using U.S. data, and includes variables such as age, education, body mass index, personal history of cancer, family history of lung cancer, COPD, smoking status, tobacco consumption, smoking duration and time since quitting. Race / ethnicity is a significant marker of lung cancer risk. Use of the PLCO_m2012_ risk prediction tool in North America identified that the model under-predicted risk for the American Indian / Alaskan Native and “Black” populations. Ethnicity adjustors were subsequently included in the model. Retrospective studies have demonstrated that selection using model-estimated risk such as the PLCO is superior to NLST-like criteria. Screening using model-estimated risk has higher sensitivity and positive predictive value (PPV), prevents more deaths and has greater cost-effectiveness than screening using NLST-like selection criteria^17^.

In the study described in this protocol, assessment of a person’s risk of developing LC will be undertaken using the PLCO_M2012_ model. On advice from Prof Tammemägi who developed the model we will use a risk threshold of 2.0%; that is, people whose 6-year probability of developing lung cancer is ≥ 2.0% will be offered lung cancer screening. People with a ≥30 pack-years smoking history will be eligible for and offered lung screening even if their risk is below the 2% threshold.

We have elected to the PLCO_M2012_ model as it has higher sensitivity, specificity, and positive predictive value when compared with the NLST criteria^9^, and has been validated in a number of countries ^18^. It was developed using U.S. data and includes age, education, body mass index, personal history of cancer, family history of LC, chronic obstructive pulmonary disease (COPD), smoking status, tobacco consumption, smoking duration and time since quitting and also includes a predictor for race/ethnic groups as it was found that race/ethnicity predicted LC independently and in addition to the other predictors in the model^19^. In particular, African American and American Indian/Alaska Native (AIAN) populations were at additional elevated risk when compared to Whites and other ethnic groups. It is important to ascertain whether the PLCO_M2012_ accurately predicts Māori risk of LC, and as the model under-estimates AIAN people’s risk of LC, it may also do so for Māori, who are over 3 times more likely to develop LC even though tobacco exposures are similar^6^. We are very interested in assessing how well the PLCO_M2012_ risk prediction model predicts actual prevalence. However, we will not have sufficient power to do this with the sample proposed in this application unless there is a very large difference between predicted and actual prevalence of LC, and this question is the focus of a further proposed study that is currently under assessment by HRC for potential funding. Until we have sufficient NZ data to examine predicted versus actual risk, a conservative approach is required to mitigate the possible underestimation of Māori risk. To this end we will use the ethnicity weighting for AIAN people when calculating risk for the Māori participants.

People with a ≥30 pack-years smoking history will be eligible for lung screening even if their PLCO_M2012_ derived risk is below the 2% threshold. Pack-year is defined as number of packs of cigarettes smoked per day multiplied by the number of years smoked. If a participant stopped smoking for 6 months or more and then restarted smoking again, the time will be subtracted from the total duration of smoking in 0.5-year increments.

In addition to using the PLCO_M2012_ model, we will also ask participants some additional questions around workplace exposure risks for lung cancer. The data collected from these questions will contribute to a wider NZ study led by Dr James McCleod at Worksafe. This study looks at risks of lung cancer related to occupational causes, which will become relatively more important over time, as smoking prevalence decreases. Data collected in our study will be shared with this group in an anonymised fashion and will not be sent overseas.

In addition, we will also ask participants about their lifetime exposure to passive smoking. This data will contribute to a future planned study of the relevance of the PLCO_M2012_ to the NZ population and how we can develop a more accurate risk model, particularly for Māori.

Following screening by Low Dose CT (LDCT), scans will be assessed for abnormalities. A key potential for harm in lung cancer screening relates to the detection of a nodules that is investigated as potentially being cancer and that subsequently is determined not to be cancer. The efficiency of lung cancer screening is helped by using optimized criteria for reporting and managing lung nodules detected by CT scanning, and our study proposes to use the Pan-Canadian Early Detection of Lung Cancer Study (PanCan) model. This model has been developed over a number of years and now includes multidimensional nodule measurements (mean diameter (PanCan-MD) or volume (PanCan-VOL)), rather than nodule diameter alone^20^. The PanCan models have been externally validated for use in the National Lung Screening Trial and are, we believe, the models that will produce the least risk of harm in our study.

### COPD

As part of this study we also plan to assess participants for COPD. In Aotearoa NZ, rates of COPD among Māori are higher than in other populations, and Māori generally have worse lung function at lower smoking exposures than New Zealand Europeans (NZE).^21^ COPD has been shown to increase the risk of lung cancer,^1,22,23^and among Māori with LC, a higher prevalence of COPD is observed at lower smoking exposures.^21^ The burden of severe COPD is higher among Māori compared to non-Māori. In 2015, COPD-related mortality among Māori aged ≥45 years was more than twice that of their non- Māori/Pacific/Asian (non-MPA) counterparts (RR 2.24 CI 2.07 – 2.42)^24^, with the greatest inequities observed among Māori women where COPD was the third leading cause of death (2010-12)^25^. An inequitable and increasing burden of non-fatal severe COPD is also borne by Māori^25,26^, the estimated age-standardised total population prevalence of severe COPD in 2017 among Māori was 2.4%, compared to 0.8% among non-MPA peoples^27^.

In Auckland DHB (ADHB) and Waitematā DHB (WDHB), the catchment area for the study, Māori aged over 45 years have 3.8-4.1 times higher hospitalisation rates for COPD than European/Other populations^28,29^. Rates of first COPD hospitalisations across New Zealand are also increasing among Māori aged ≥45 years and decreasing among non-MPA peoples, with the age-standardised RR among Māori rising from more than 2.5 times higher in 2000 to over 3.5 times higher in 2017^24^. The upward trend in Māori hospitalisation rates will continue while the prevalence of smoking among Māori remains relatively high. Māori hospitalisation rates increase from 40 years of age onwards, ten years earlier than the non-Māori population^26^. This difference may reflect earlier onset of disease, more severe disease at an earlier age, inequities in the quality of healthcare resulting in higher morbidity among Māori, or a combination of all three factors.

In Aotearoa NZ, COPD is under-diagnosed, particularly early in the disease course, but the degree of current unmet need among the high-risk Māori population is unknown. While some information about the prevalence of severe COPD can be gathered from those who are hospitalised for the disease, little is known about the true burden of COPD in the community among those with milder forms of the illness. In primary care, COPD is under-diagnosed and therefore under-treated.^1,2,30-34^ This is due to a combination of factors, including the non-specific symptoms,^2,3,27^ slow onset of disease and lack of systematic consideration.^35^ There is a dearth of evidence about the extent to which COPD is under-diagnosed in primary care.

There are some clear criteria for how to diagnose and assess COPD severity. A diagnosis of COPD should be considered in patients with symptoms suggesting COPD (dyspnea, cough, sputum, wheeze, recurrent lower respiratory tract infections) and/or a history of exposure to risk factors for the disease, and confirmed by spirometry results consistent with persistent airflow limitation^1,36^. The severity of airflow limitation can be categorised into mild, moderate, severe and very severe. Although spirometry is used to diagnose and assess the severity of airflow obstruction, further assessment of symptoms is needed. The COPD Assessment Test (CAT) is an 8 item, validated tool that quantifies symptom burden and impact on wellbeing and daily life. It is recommended for, and widely used, in routine practice^1,37^. Electronic and paper versions are available^37,38^. CAT scores range from 0 to 40.

Our study provides a feasible and efficient opportunity for diagnosing and assessing COPD severity among study participants. There are a number of key barriers^22^ to COPD diagnosis in primary care including the availability, training requirements and quality of spirometry. Including COPD in LCS programmes is an opportunity to improve the diagnosis and management of COPD. Two recent LCS programmes incorporating routine spirometry for individuals of predominantly lower socio-economic status in the United Kingdom reported that between 37-57% of eligible screening participants had airflow limitation consistent with COPD, and 50-70% of those were undiagnosed^39,40^. Among the 2,541 current or former smokers aged 55-74 years residing in three deprived areas of Manchester who were involved in the community-based LCS programme undertaken in that city, spirometry was successfully performed among more than 99% of attendees^40^. Furthermore, in the treatment arm of the Lung Screen Uptake Trial undertaken in deprived areas of London among current or former smokers aged 60-75 years, spirometry was performed in around 98% of participants^39^. There are other international LCS studies that have incorporated spirometry for less high-risk populations. The nested sub-study of COPD within the LCS trial is likely to further improve the cost-effectiveness of the screening pathway.

Once diagnosed, good management of COPD in primary care is important. Best practice management of COPD can slow progression, prevent exacerbations, and mitigate the impacts of this condition^1,33,41,42^. The key features of high-quality evidence-based COPD primary care are: 1) diagnosis through spirometry accompanied by assessment of the severity of COPD symptoms; 2) pharmacotherapy according to the severity of the COPD with the goal of maximising the person’s function together with regular re-assessment of COPD severity and review of medications; 3) prevention of acute exacerbations and early management of acute exacerbations when they occur; 4) non-pharmacological strategies such as the provision of COPD action plans and strategies for managing dyspnoea, self-management education and support, encouraging exercise, referral for pulmonary rehabilitation, and referral to community support groups; 5) smoking cessation support for those that continue to smoke; and 6) maximising management of co-morbid conditions such as cardiovascular disease, diabetes, anxiety and depression^43^.

Currently, primary care management of COPD is sub-optimal. This sub-study will provide valuable insight into whether systematically assessing COPD as part of a LCS pathway leads to optimised management of COPD within a high-risk population. When standardised COPD assessment results are returned to primary care, practitioners will also receive guideline-based management recommendations for COPD. This study will evaluate whether new COPD patients are treated as recommended and whether existing COPD patients’ care is optimised.

## Research Programme

Our research programme, Te Oranga Pūkahukahu (The Lung Health Check) aims to provide directly policy relevant information to guide the implementation of lung cancer screening nationally. The research programme is intentionally designed for Māori, aiming to reduce Māori mortality, and the life expectancy gap, from lung cancer.

To date the programme has focussed on three key pieces of work:

1. A survey alongside a series of focus groups to address important gaps in our understanding about how Māori would like to engage with a screening programme for lung cancer, and how the programme should be designed to reduce inequities. These findings have been incorporated into the design of this trial and a paper is currently in preparation.
2. A re-analysis of the cost effectiveness of lung cancer screening in NZ to address issues in a 2018 study from Jaine et al^44^ which concluded that LCS was unlikely to be cost-effective for any group in New Zealand. Jaine et al’s conclusions hampered progress in national discussions on lung cancer screening. Furthermore, more up-to-date data for some parameters in the model were available and the original authors did not undertake any equity-focused analyses. As a result of these issues, we undertook to update the cost-effectiveness modelling, using the original model. Our re-analysis found a number of issues with the model which altered the findings of the original study. Once these issues were corrected, we updated a range of parameters with the most recently available evidence and extended the model with alternative assumptions based on Māori health equity. Our resulting paper demonstrated that lung cancer screening is likely to be highly cost-effective for all populations groups, particularly for Māori women^45^.
3. Trialling lung cancer screening in New Zealand, initially Māori-specific.

## Aims and objectives

This study will focus on testing core invitation methods:

- a primary care practice based invitation and risk assessment process
- a DHB based centralised invitation and risk assessment process.

In this trial, we hypothesise that the invitation to lung screening conducted in primary care will result in higher levels of participation by Māori than a central hub with established expertise in conducting screening programmes and skills in active follow-up of Māori. This study is focused on:

- Determining the effectiveness of two lung cancer screening invitation strategies; primary care vs central hub
- Describing key lung cancer screening parameters to inform a potential national programme in NZ
- Testing the performance of the risk prediction tool
- Evaluating contextual implementation factors in order to optimise a future NZ screening programme, including incorporating COPD assessment

This is a Māori-specific study. It is the intention that, by identifying the most successful invitation methods for Māori, a successful screening program can be built which will allow detection of curable lung cancer. This in turn will reduce mortality, improve chances of cure, and substantially reduce health care costs in New Zealand. Current adult cancer screening programmes fail to deliver equitable outcomes for Māori. In order to avoid a LCS programme having similar inequitable outcomes we must design the programme so that it works well for Māori in the first instance.

In addition to the overall aims, we intend to test some of the key assumptions used in the sample size calculations and to test the planned processes.

Key questions that we will address include:

### Pilot phase

For the first group, trial participants will include DHB kaumatua, Te Hā Kotahi members and their whānau, and up to four primary care practices. This phase of the study will result in up to 50 scans, and will test key trial processes, including:

- Usability of the risk assessment tool
- Data collection and IT system
- Invitation materials
- Consent processes
- Results management
  - Reporting in private community radiology clinic
  - Standardised screen read and reporting
  - Use of volumetric analysis
  - Incorporation of blood biomarker analysis
  - Interpretation of results by providers and experience of providing results to participants and whānau
  - Interface between primary and hospital care for positive screen management

### Full trial

#### Primary outcome:

The full trial including a further 500 scans of eligible participants will address the primary research question of whether an invitation to lung screening conducted in primary care results in higher levels of participation by Māori than a central hub-based invitation method.

#### Secondary outcomes:

In addition, the trial will include the assessment of outcomes needed for the development of a national screening programme, including:

Participants and whānau experience of LCS:

- What is the participant and provider experience of LCS?
- What is the experience and acceptability of the shared decision making and results management processes as outlined in the protocol?
- What is the participant burden using the tools outlined in the study methods?
- What is the provider burden using the processes outlined in the GP clinic based arm of the study?

Parameters relating to recruitment and participation in the LCS pathway:

- What is the accuracy of recording of smoking history in general practice among those who are identified as potentially eligible for risk assessment i.e. those who are not recorded as ‘never smokers’?
- What % of Māori aged 55-74 in a general practice are potentially eligible for lung cancer screening
- What % of potentially eligible people agree to undergo risk assessment
- What % of those undergoing risk assessment are eligible for lung cancer screening
- What % of those eligible for lung cancer screening decide to proceed with screening
- What % of those who decide to proceed with screening attend their appointment for screening
- We have also included in our risk assessment a question about work place related exposure risks for lung cancer. The data from this question will contribute to a wider NZ study of lung cancer related to work place exposure.
- In addition, we will also ask participants about their lifetime exposure to passive smoking, using questions taken from the Lung Health Questionnaire developed for the International Lung Screening Trial (ILST). This data is collected using a standard validated scale, used in ILST and European trials, and will contribute to a future planned study of the relevance of the PLCO_M2012_ to the NZ population and how we can develop a more accurate risk model, particularly for Māori.

Parameters relating to the assessment of COPD within LCS:

- What is the prevalence of COPD among participants who proceed to CT scanning
- What are the characteristics of COPD in those assessed and diagnosed
- How are participants with COPD managed within primary care following this assessment
- How do GPs change the way they manage COPD after they are provided with COPD results and management recommendations
- What is the participant experience of COPD assessment
- How do participants view the inclusion of COPD assessment in future LCS programmes

## Study Design

A cluster randomised trial of two invitation methods to participate in LCS. The two invitation pathways will be tested end to end (invitation, risk assessment, shared decision making and CT scan).

Participating practices will be randomised (1:1) to either the primary care led or central hub arm. The randomization sequence is computer generated at the start of the study using a random number generation formula. For each pair of practices recruited, a sealed envelope containing an instruction that either the first or the second in the pair is to be placed in the primary care-led arm is opened by the programme manager. There are equal numbers of envelopes with each instruction and the envelopes are randomly sorted). Pairwise randomization ensures that there are equal numbers of practices in each group and it means that not all practices need to be recruited from the outset, a distinct advantage when recruiting primary care practices in the current, Covid-constrained environment.

Within the overall randomised trial of lung cancer screening methods will be a nested cohort study. In this sub-study, participants (those eligible for a CT scan) will be assessed for COPD.

### Study setting/Location

The project will be hosted at Waitematā District Health Board (WDHB) and Auckland District Health Board (ADHB), in both primary care and hospital based facilities. We anticipate that we will initially recruit between 2 and 6 primary care practices in order to test key assumptions prior to continuing the study in the full cohort of up to a further 48 practices (24 in each study arm) (Figure 2). The CT scans will be conducted in private community fixed-site radiology facilities located centrally.


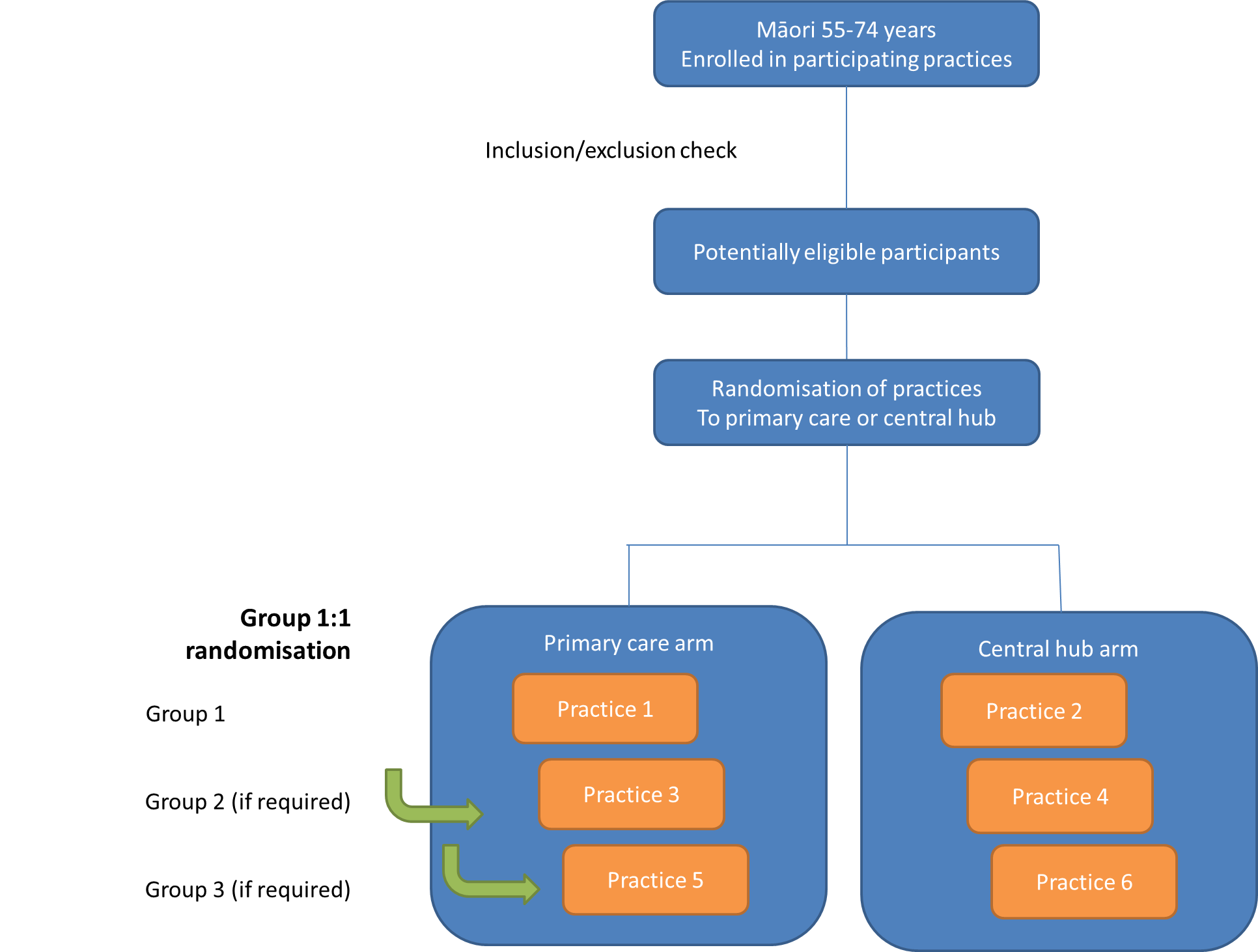


Figure 2: Practice Randomisation schematic (the groups will extend until we reach the required number of practices – up to a maximum of 48 (excluding pilot practices))

### Study Population

The initial study population includes all Māori between the ages of 55 and 74 who are not recorded as “never smokers” in the practice management system.

### Eligibility Criteria

#### Patient eligibility criteria:

- Recorded as Māori in the primary care enrolment
- Enrolled in participating general practices
- Able to provide informed consent to participate
- Agree to undergo the activities required for participation in the trial

#### Patient exclusion criteria:

- Never-smokers
- Previous diagnosis of lung cancer
- Have other non-curatively treated cancer outside the lung
- Clinical symptoms suspicious for lung cancer e.g. haemoptysis, chest pain, and weight loss
- Have received chemotherapy or cytotoxic drugs within the last 6 months
- Documentation of a chest CT within two years (this will be initially checked by asking the participant if they have had such a scan, followed by a review of records to confirm)
- Pregnancy
- Unable to provide informed consent due to cognitive problems
- Unwilling to provide consent
- Additionally, for the assessment of COPD, if participants are unwilling or unable (due to a comorbid disease) to undergo spirometry testing they will be excluded from this part of the study.

### Deferrals

- Pneumonia or bronchitis requiring antibiotic treatment within the last 12 weeks

### Study Procedures/Methods

The overall study schematic is outlined in Figure 3.

### Recruitment of participants

#### Identification of potentially eligible participants

Permission will be requested from PHOs to access primary care enrolment data initially. The PHOs of practices who agree to participate in the study will compile lists of potentially eligible Māori, aged 55-74 who are not recorded as “never smokers”. We will request a waiver of consent from HDEC to allow access to these data.

Participating practices will conduct an initial review of the potentially eligible participant list and assess for inclusion and exclusion criteria and refine the initial list.

Practices will provide the study team with the finalised lists that include demographic information, contact details, relevant medical history, and recorded smoking status including all excluded participants with reasons for exclusion.

#### Randomisation of practices

General practices will be randomised at a 1:1 ratio to either the primary care or centralised arm of the trial. This will reduce the risk of contamination if patients were the unit of randomisation and patients in both arms of the trial were located in the same practice.


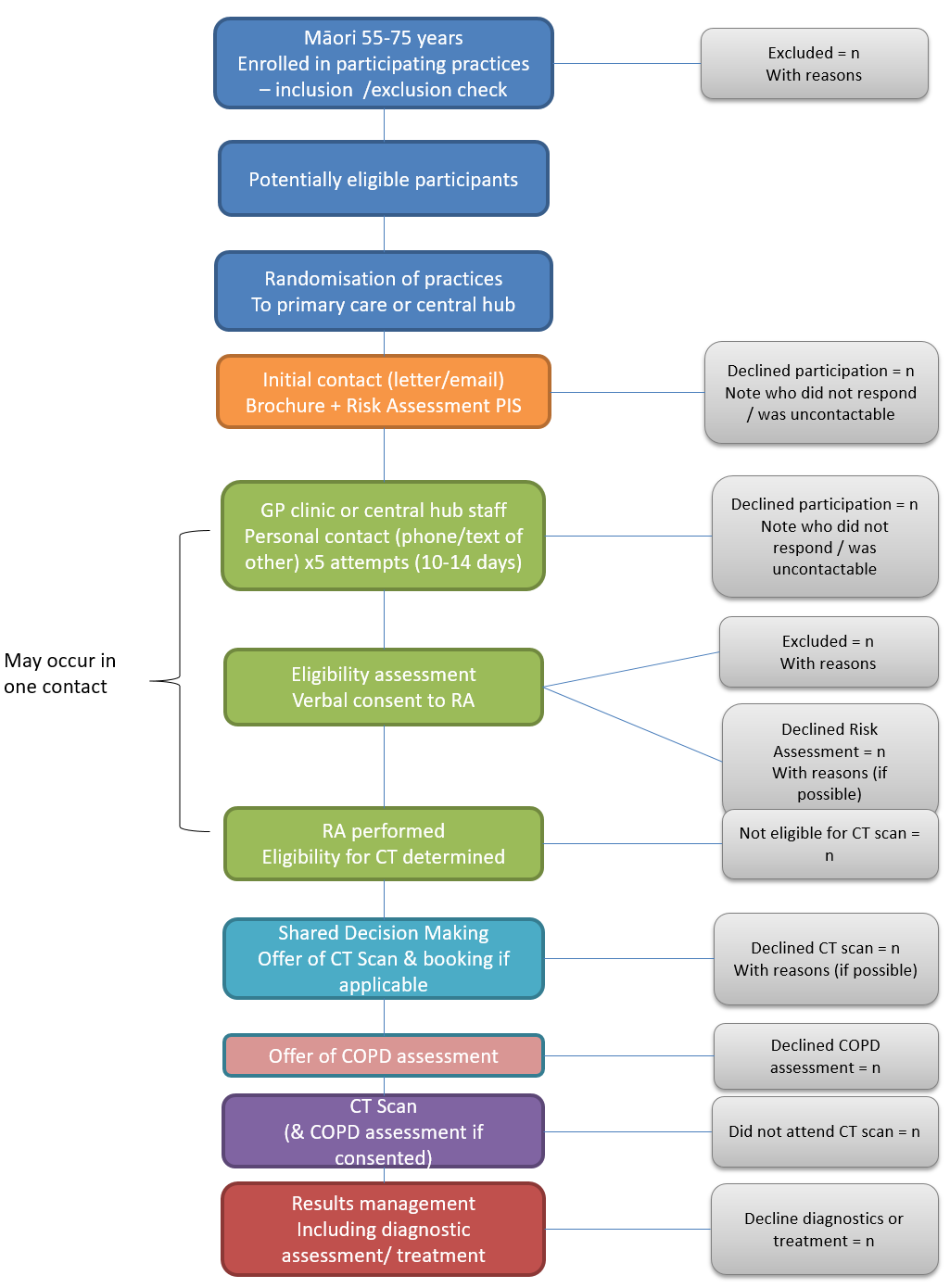


Figure 3: Study schematic

#### Invitation to participate

Potentially eligible people will be sent an email, letter or text with a link to study information, advising them that they will be contacted about participating in the trial and COPD assessment, including an overview brochure and a participant information sheet outlining the risk assessment process. For participants in the central hub arm, this will include a phone number they can use to contact the central hub to request further information, decline participation, or proactively begin participation. Participants in the GP arm, will be advised to contact their GP regarding any possible questions. They may opt out of any further contact if they wish at this point.

After a 10 to 14-day period participants will be contacted by phone and invited to participate in the trial by a nurse or other clinic staff (GP clinic arm), or by a staff member of the central hub (see Figure 3). Five attempts to contact potentially eligible participants from the list will be made, thorough a variety of methods (email, phone, letter, text). In both arms, phone calls and texts will be made at different times of day (e.g. daytime and evenings). Based on our prior equity-focused research we have found additional contact attempts were important to support participation in the studies. Contact attempts will be captured in the study IT system. Declining to participate will result in no further contact about this study.

The purpose of the contact is to explain the trial, if interested, and then to confirm inclusion/exclusion criteria. For eligible participants, we will then seek verbal consent to participate in the risk assessment. Potential participants will be sent the full Risk Assessment Participant Information Sheet, and will be given sufficient time to read it and ask questions.

In the primary care-based arm clinic staff will undertake this process. Primary care arm staff will also be able to engage with people on the list opportunistically, if they attend the practice for another reason.

In both arms, staff involved in this invitation procedure will be given training and a procedure manual to ensure that trial procedures are standardised.

#### Whānau involvement

Whānau or other support people are both invited and welcomed to participate in all of the parts of the lung cancer screening processes and COPD assessment – from invitation, risk assessment, shared decision making, CT scan through to management of results.

#### Risk Assessment for Prediction of Lung Cancer Risk

The risk assessment will be done by practice nurses (primary care arm) or the project research nurse (central arm) in-person or over the phone depending on patient preference. A standardised protocol for completing the risk assessment will be used; staff in both arms of the trial will receive training in this and a procedure manual will be provided to everyone undertaking these assessments. Whānau or other support people can participate in the risk assessment process.

The PLCO_M2012_ risk prediction tool will be used to assess a participant’s risk of lung cancer. Participants with a $\geq2 \%$ risk of developing lung cancer within six years will be eligible for a CT scan.

People with a ≥30 pack-years smoking history will be eligible for lung screening even if their PLCO_M2012_ derived risk is below the 2% threshold. Pack-year is defined as number of packs of cigarettes smoked per day multiplied by the number of years smoked. If a participant stopped smoking for 6 months or more and then restarted smoking again, the time will be subtracted from the total duration of smoking in 0.5-year increments.

#### Shared Decision Making and the offer of lung cancer screening

Participants who meet risk threshold or criteria based eligibility for LCS will be offered a Shared Decision Making process. This process will provide them with a decision aid, which will contain information about screening in general, and lung cancer screening in particular (including risks and benefits, possible findings, follow up of CT scan findings etc.) allowing them to make an informed decision about whether to proceed with screening. Whānau are welcome and invited to participate at all stages of the Shared Decision Making process. If they choose to proceed, written consent will be obtained and an appointment will be made for them to undergo the scan. The shared decision making process can be undertaken in-person, by phone or using a digital platform (secure telehealth platform) immediately following the risk assessment or at a later date, according to the person’s preference. Where people opt for a phone or digital platform based decision making process we will be able to provide them with the same information and resources used in an in-person process. The study team has developed a shared decision making guide, and adapted a decision aid supported by health literacy experts, which is included in Appendix 4.

#### Offer to participate in COPD assessment

Participants who are eligible for LCS (by LDCT) will also receive written information about COPD assessment. After they have undergone Shared Decision Making for LCS, the study nurse will explain the COPD assessment and offer them the opportunity to participate. They will be asked for their written consent to proceed with COPD assessment.

### CT scan

Scans will be undertaken at certified private community radiology providers who will operate equipment which meets the minimum specification for dose and slice thickness parameters for low dose lung screening CT examinations.

The radiology provider will provide a summary report on the imaging stating that the scan was conducted for research, and the scan will then be sent to the research radiologist where it will be reviewed by them or their registrar within a few days of the scan being conducted.

The images will be pulled as thin section CT data to ADHB and WDHB where the images will be reviewed by dedicated named radiologists who will complete a standardised proforma report of findings of interest to the study including: lung nodules, structural lung disease e.g. emphysema and interstitial lung disease, significant airways disease e.g. bronchiectasis, and other incidental findings. Where nodules are present, lung nodule volumetry will be performed and recorded in the report. The report will be sent to the participant’s GP, the study respiratory physician and the study co-ordinator. CT scan result management and follow up is detailed below.

### COPD assessment

The COPD assessment nurses will meet the participants at the radiology centre when they attend for their CT scan and the COPD assessment will be undertaken after the CT scan. This will include assessment of the participant’s COPD-related symptoms (using the COPD Assessment Test) and spirometry to determine the presence and extent of airflow limitation. A CAT score $>$ 10 will be used as the threshold for regular treatment. Spirometry will be performed using a portable spirometer (Easy-One^TM^; ndd Medizintechnik AG, Zurich, Switzerland) using the standardised process recommended by the New Zealand Medical Association^63^ with the highest value of the best three acceptable blows used for classification of COPD status. A forced expiratory volume (FEV1) to forced vital capacity (FVC) ratio of less than 70% of the expected result for that participant’s age and sex (FEV1/FVC < 0.70) is diagnostic of airflow limitation (COPD)^1,36^. These FEV1 results are then categorised according to NZ guidelines Following the first test, the spirometry will be repeated using a bronchodilator in order to assess COPD severity.

This will be undertaken using a standardised protocol by Māori nurses trained in undertaking spirometry and the study procedures.

Data will be collected from the participant (spirometry, symptom assessment, patient-reported diagnosis, medications and health service utilisation), GP and hospital records (COPD and related classifications, COPD management and health service utilisation in the 12 months prior to assessment), and the LCS trial (demographics, quality of life, smoking, comorbidities).

Participants will also be asked to complete a post-COPD assessment evaluation questionnaire (Appendix 16)

### Nodule assessment, classification and management

A variety of nodules, with different levels of risk of actually being cancer, are expected to be identified by the lung CT scan. All identified nodules will require follow up and further consideration. Diagnostic assessment, such as bronchoscopy or biopsy, used in the investigation of nodules have potential harms for the patient. These harms can be minimised by minimising the number of diagnostic procedures performed on nodules that are not cancer. This can be achieved through the use of cancer risk thresholds and repeat short interval scans (with serial volumetric assessment). Interval scans may be 3, 6 or 12 months as determined by the nodule risk. Nodule risk is based on nodule type and size, and is used to guide subsequent actions / investigations (Table 1). A number of tools are available and are currently used within DHBs in New Zealand to identify which nodules need further investigation, helping to reduce the risk of harm from invasive investigations. In this study we will use the PanCan nodule risk management classification system to determine the malignancy risk of a nodule and determine recommended follow-up of the scan. The PanCan system has been used in international LCS studies including our Australian colleagues undertaking the International Lung Screening Trial (ILST). Table 1 below describes the nodule categories of risk for malignancy.

Table 1: PanCan nodule malignancy risk and associated actions for screening scan.

| **Category** | **PanCan nodule malignancy risk at screening scan** | **Action** |
| --- | --- | --- |
| 1 | No significant nodules: normal finding, nodule risk < 1.5% | No further action |
| 2 | Low risk: nodule risk 1.5% to < 6% | Repeat CT scan at 12 months (follow up scan)  No growth at 12m: repeat in further 12m and, if no growth then no further action*.  Any interval growth at 12 m: refer for diagnosis |
| 3 | Moderate risk: nodule risk 6% to < 30% | Repeat CT scan at 3m (follow up scan)  3m scan result: No growth: repeat scan at 12m intervals until no growth for 2 years and then no further action  If interval growth at 3m or any of the subsequent scans: refer for diagnosis  Consider referral for diagnosis if nodule risk 10 to < 30% |
| 4 | High risk: nodule risk $\geq$ 30% | Refer for diagnosis |
| 5 | Suspicious: mass/lesion; mediastinal or hilar lymphadenopathy irrespective of nodule size | Refer for diagnosis |

*at the radiologist’s discretion, follow up may be extended for Ground glass opacifications as these are slower growing

Nodules will be characterised by site (lobe, juxta-pleural, perifissural), volume, density and presence or absence of spiculation or a benign pattern of calcification and nodule type (solid (SN), part-solid nodules (PSN) or pure ground glass nodules (pGGN)). Solid nodules with benign features, popcorn calcification, and intrapulmonary lymph nodes will be included as a positive scan.

The total number of nodules and other findings will be recorded. Where multiple nodules are detected, at least two, including the largest, will be characterised. Low and moderate risk nodules identified on the screening scan will have follow up scans to assess growth. New nodules detected on a follow up scan are managed differently to pre-existing nodules as there are significantly different implications for being a LC. Additionally, PSNs are also more likely to be malignant.

Blood biomarker analysis

Participants will be asked to donate blood sample for blood biomarker analysis at the time of their CT scan, which will be taken by a study nurse qualified in phlebotomy. As described above, biomarker analysis has been proposed to increase the specificity of LDCT screening. This study will assess the implementation of multiple types of blood biomarker. From each patient: (i) a blood sample of 10mL will be collected into a specialised cell-free DNA collection tube from which plasma will be extracted for downstream ctDNA analysis (see below), (ii) 10mL will be collected into an EDTA tube for complete blood count, and (iii) 10ml collected into a serum separator collection tube for protein biomarker testing. All three tubes will be collected in the same single blood draw.

Cell-free DNA will be extracted from plasma following best practice guidelines^59^, before ctDNA analysis is undertaken using a DNA sequencing panel optimised to identify mutations in blood from primary and metastatic lung neoplasias using Ion ampliseq DNA sequencing technology. Based on variant allele frequency in the blood, DNA variants likely to originate from the patient’s germline will not be examined, by filtering out known common germline variants and/or variants present in blood at high frequency (>30%) consistent with germline origin. In this way, blood DNA variants likely to originate from the patient’s germline will be neither identified nor reported to the patient. The remaining DNA variants, which putatively originate from a neoplasia (ctDNA) will be curated against DNA variant databases which may identify their known occurrence in cancer and may also potentially suggest a likely tumour cell type of origin and may in some cases be ctDNA variants recognized as prognostic or predictive of therapeutic response. Some ctDNA variants identified, in particular those judged to have clinical actionability, may be confirmed using droplet digital PCR (ddPCR) technology in a clinically accredited test environment.

A range of non-genomic biomarkers may also be examined in blood serum, including CEA, CA125 and autoantibodies, using standard clinical procedures. Finally, the extent and impact of tobacco consumption will be estimated using one or more plasma DNA analysis methods, including: ddPCR assessment of the degree of hypo-methylation of CpG island cg05575921 in the promoter of the aryl hydrocarbon receptor repressor (*AHRR*) gene^60^. Correction will be made for patient-specific ratios of blood cell types using the complete blood count information. In up to six samples, the results of ddPCR-based *AHRR* gene hypo-methylation assessment will be confirmed using alternative analysis methods including IIlumina Infinium Methylation Beadchips^61^. As with ctDNA sequencing, germline DNA sequence data, which can be obtained from these Beadchips through deliberate analysis, will be neither obtained nor reported to the patient.

For individuals who have a lung nodule identified by CT scan (See Table 1, PanCan categories 2-5), ctDNA, protein biomarker analysis (if undertaken) and complete blood count will be analysed in an identifiable manner, to facilitate feedback of incidental findings if actionable ctDNA mutations were identified (see below). All remaining individuals, who have not had a lung nodule identified by CT scan, will have ctDNA, protein biomarker analysis (if undertaken) and complete blood count analysed after all patient samples are collected. These results will not be examined in an identifiable manner, rather, the blood biomarker and CT scan results for all individuals irrespective of whether they had a lung nodule identified by CT scan will anonymised before pooling for final analysis. Concordance between LDCT and biomarker results will be estimated and the marginal benefit of plasma biomarker data on the sensitivity of LDCT screening estimated using regression approaches, as we have undertaken previously^62^.

#### Incidental findings

Incidental findings are defined for the purposes of this study as any abnormalities or diseases identified on the CT scan other than lung cancer or lung nodules. It is expected that screening will detect incidental findings. Table 2 outlines categories of incidental findings, required action and follow-up to ensure action was undertaken.

Table 2: Categorisation of incidental findings, actions required and follow up

| **Category** | **Required action** | **Follow up** |
| --- | --- | --- |
| 1: major findings that may be life threatening and direct referral to hospital is required | Direct referral to hospital by radiologist or, if the participant is not on-site the radiologist will call the participant’s GP | Research nurse to call radiologist/GP to ensure referral done immediately |
| 2: significant findings (e.g. significantly dilated aortic aneurysm) requiring urgent referral | Urgent referral by participant’s GP | Research nurse to liaise with GP to ensure referral done within two weeks after report is issued |
| 3: findings indicative of cancer at another site (e.g. breast) or ctDNA information, after validation in a clinical grade test, requiring urgent referral; | Urgent referral by participant’s GP in liaison with oncology / respiratory specialists on project team. |  |
| 4: other non-cancer findings requiring non-urgent referral to primary or secondary care (e.g. significant fibrotic interstitial lung disease, TB, bronchiectasis) | Treatment by GP or non-urgent referral (by GP) to tertiary care | Study nurse to follow up on referrals in Éclair one month after report is issued |
| 5: non-cancer findings that may require management in primary care (e.g. COPD, ectatic aorta, coronary calcification) | Management by GP | Study co-ordinator to call GP to ensure patient has been seen |
| 6: findings that are usually not directly associated with a beneficial intervention (e.g. bronchial wall thickening) | No management required |  |

#### Biomarker-related incidental findings

Potential incidental findings identified via biomarker analysis will be reviewed by an external advisory group set up by Professor Print. This group is a Māori advisory group which already advises Prof Print on other projects and will be leveraged to also advise on this project, particularly around the interpretation of results and whether incidental findings are significant enough to be fed back to participants. Should any results from this study need to be discussed, the study PI or their delegate will also attend. Any actionable finding will be dealt with by the respiratory and oncology specialists in the research team, who will also provide support to the participants GP as needed.

### CT scan result management and follow up

#### Communication to participants and their whānau

In the GP arm, the GP or practice nurse will discuss the results of the scan with the participant and their whānau. For participants with clear or negative scans, this will involve a phone call from the nurse. With more complex results, the participants will be invited back to the practice for a discussion with the GP (at no cost to them). Should further scans or diagnostic investigations be required, the GP will refer the participant to the relevant tertiary service.

In the centralised arm, if no follow up is required, then the study nurse will contact the participant to inform them of the result. For participants with positive scans, the participant’s GP will contact the participant to arrange an appointment to discuss their results, and to arrange follow up (interval) CT scans and referrals for diagnostic investigations, with support from the study respiratory physician.

In both arms, participants will again be taken through their options, using a shared decision making process. Participants will always have a choice in the types of investigations they undertake and / or treatment they receive and are able to choose to decline if they wish.

#### Referrals

In both arms the participants’ scan results will be sent to the GP practice, and the study co-ordinator. Positive scans will also be sent to the study respiratory physician. In all reports, the screening radiologist will clearly categorise the scan, including recommendations for follow up and who is responsible for each action (Table 1). The report will also identify who is responsible for arranging any required follow up.

In both arms, positive scan results will be communicated to participants via their GP and, where required, the study respiratory physician. The study co-ordinator will follow up with the GP and with the study respiratory physician two weeks after receiving the report to ensure the patient has been informed of the scan result and any required follow up arrangements instituted.

Should further investigation be required, then the study respiratory physician will contact the participant to arrange appropriate follow up (Figure 3). The study co-ordinator will follow up with the study respiratory physician two weeks after receiving the report to ensure the patient has been informed of the scan result and any required follow up arrangements instituted.

Participants requiring follow up CT scans and diagnostic investigations will be referred by the respiratory physician to the relevant DHB services for ongoing care. People who are diagnosed with lung cancer will be referred to the Multi-Disciplinary Team of their DHB of domicile.

Timelines for all above procedures and discussions / referrals will be recorded and monitored by the study team to ensure timely follow up of participants and to feed into the study analysis.

### Results of COPD assessment

#### Participants

At the end of the assessment the nurse will discuss the spirometry results, CAT score and GOLD classification with the participant and their whānau. Where the results are consistent with a diagnosis of COPD the nurse will provide the participant and whānau with information about COPD and its management, including the health benefits of diagnosis and good quality management (reduced symptoms and progression of COPD) and the opportunities for increased participant and whānau self-management and autonomy over the condition. The nurse will use standardised resources developed specifically for the study. The resources will incorporate design features that support the development of participant’s/whānau COPD-related health literacy. All participants who are current smokers will be provided with brief advice and offered help to quit smoking after they have discussed the results of their COPD assessment with the nurse. The nurse will follow up with the participant/whānau 1-2 weeks after the assessment to ascertain how they are feeling about the diagnosis and discuss any questions or issues that have arisen since the assessment.

#### Participant’s GP

The GP will be sent a standardised letter including the measured FVC and measured FEV1, (with calculated % of predicted value) and the FEV/FVC ratio. The GOLD classification of severity (GOLD A (mild), B (moderate), C (severe) or D (very severe)) will be included in the letter. In addition the participant’s CAT score with an explanation of the participant’s score, and COPD-X guideline management recommendations for the participant’s GOLD classification will be included in the letter. Non-COPD related findings observed on spirometry (e.g. restrictive airflow) will also be reported on the letter to GPs.

#### Referral for specialist assessment

In addition to the provision of information to their GP, the ADHB COPD health pathway will be used to determine if participants with moderate or severe COPD (GOLD classification B, C and D) should be referred for specialist assessment at their local respiratory clinic. Dr Hotu will review results for all participants with GOLD B, C, or D classifications to confirm if referral is required.

### Results of biomarker study

The collection of blood and analysis of biomarkers is, at this stage, a feasibility study to assess how this might be built in to a future lung cancer screening programme. At this stage, we do not anticipate the analysis of the blood will be done in “real time”and we anticipate the potential for any incidental findings will be low. However, should any results be considered important, these will be reviewed by a Māori expert advisory group who will comment on whether they believe the findings to be clinically actionable. Any such results will also be reviewed by the lung cancer screening study Steering Group and Technical Advisory Group, and should there be agreement, actionable results will be shared with the patients general practitioner, who will be provided with support as to how these results will be communicated.

### Secondary research questions

As part of the study, we will also seek to determine the answers to our secondary research questions, including information on participant and provider burden, and the experience of participants at different stages of the LCS process.

Although the primary focus for this study is on testing invitation strategies, the effectiveness of LCS pathways has not yet been determined for any indigenous population including Māori. Therefore, we plan to assess our approach using an “Effectiveness-Implementation Hybrid Type II design”^47^, using both the RE-AIM^48^ framework planning questions and a locally developed kaupapa Māori implementation framework *He Pikinga Waiora*^49^. This framework has indigenous self-determination at its core and includes: cultural centredness, community engagement, systems thinking, and integrated knowledge translation (Table 3).

Key qualitative questions we wish to address include answers to the following questions:

- What is the participant and provider experience of LCS and COPD assessment
- What is the experience and acceptability of the shared decision making and results management processes as outlined in the protocol?
- What is the participant burden using the tools outlined in the study methods?
- What is the provider burden using the processes outlined in the GP clinic based arm of the study?
- What are the barriers and facilitators for participating in COPD assessment?
- How do the COPD assessment and its findings impact the participant and whānau?
- What are participant views about whether COPD should be included in future LCS?
- What are participant views around blood biomarker analysis?
- What percentage of participants offered biomarker analysis accept the offer?
- What is the feasibility / patient experience of taking blood in a single visit alongside a CT scan and spirometry?

These will be addressed using the questionnaires included in Appendices 8-17, alongside focus groups, interviews and feedback from primary care providers. Our approach will record feedback and experiences of participants (and non-participants) and providers, allowing us to incorporate this into future refinement of the process.

Our previous focus groups and surveys have fed into the development of this study, in fact our primary research question was generated from our survey finding of equipoise between the potential invitation strategies. Determining real world effectiveness of different invitation methods is therefore a key area of focus.

After the initial 50 participants have been scanned, we will review participant and stakeholders experience to assess acceptability and appropriateness of the process and to improve feasibility. We will ensure that feedback from both primary care partners and hospital partners is incorporated to minimise workflow impacts. We have localised a Canadian readiness assessment tool^50^ to prepare for potential service impacts (radiology, pathology and respiratory outpatient clinics). This will be conducted prior to the trial commencing to ensure that the trial does not have significant negative impact on participating services. Stakeholder feedback will be recorded throughout the trial.

#### COPD assessment – qualitative review

Following the COPD assessment, a qualitative assessment of participants’ experiences will be conducted and we will also seek their views on the inclusion of COPD assessment in future LCS programmes.

All COPD assessment participants will be asked to complete a short survey, and, in addition, they will be asked if they would like to be contacted to discuss their experience (e.g. positive, negative, facilitators and or barriers) related to the COPD assessment process in more depth. Patient information will then be collated (name, age, gender, contact details, health alerts, date and location of COPD assessment) and stored on a password protected database separately from all other LCS and COPD assessment study information. The information will then be supplied in a password protected spreadsheet to Dr Rachel Brown (Te Atiawa/Kai Tahu) – National Hauora Coalition, who will lead this part of the study.

Participants will be contacted and invited to share their experience through participation in interviews. The study anticipates 50 whānau will be interviewed using the guide included in Appendix 17. The participants will be stratified on: gender, age and location (residential and locality of assessment) to ensure a representative sample of the population is interviewed.

This qualitative component is underpinned by Kaupapa Māori research principles, which consider a Māori world view and Māori ways of knowing, being and doing. As well, it centres Māori aspirations and equitable health outcomes. The qualitative component will support research skill development of a Māori research assistant.

Participants who consent to being contacted to discuss their experience of the COPD assessment process will be eligible for this component of the study. If the participant wishes, whānau members will be welcome to participate in the interview. We envisage 10% of those screened and assessed (n=50) COPD participants will be contacted and interviewed within a 6-week (to cater for recall and richness of experience) period of undergoing their COPD assessment. Semi-structured in-depth interviews will be undertaken using a method of the participant’s choosing (face-to-face, phone, zoom or similar digital conferencing) as well as preference for support, time and location (home, place of work, local café). Interviews will be recorded with participant consent. An interview schedule will be used to guide the discussion. It is anticipated that interviews will take approximately 30-60 minutes; however, this will be guided by the participant at the time.

All care will be taken to ensure health and safety of participants and interviewers (e.g. phone interviews, mask wearing and distance if face to face occurs, sanitising). A koha of $25 will be offered post interview in recognition of participant time and knowledge. The interviews will be transcribed verbatim and deidentified. The transcriber will sign a confidentiality agreement and interview transcripts will be reviewed by the interviewer to ensure correct information was captured and stored in an electronic file at National Hauora Coalition that will be password protected. These will only be accessible to the research team.

Once transcribed, the research team will independently code the de-identified transcripts. From this the research team will meet and using the give-way rule, coding the transcripts by consensus to create a coding framework. The give way rule pre-empts and acknowledges that there are different interpretations of research depending on worldview; where there are differing interpretations, the decision will be given to a senior researcher who holds Māori experience – so that the final interpretation of an idea or scenario is given to a Māori team member (Naepi, 2015). This coding framework will be reviewed by te Rōpū Rangahau at NHC; which is comprised of NHC kaimahi, from a variety of backgrounds; to ensure the framework is appropriate.

The framework will then be used in collaborative analysis; where research staff discuss each transcript together to jointly agree on an interpretation against the coding framework. The raw findings will then be presented to te Rōpū Rangahau, where feedback will contribute to a refined coding for the transcript. This process will be used for each transcript to ensure robust interpretation. Once each transcript is coded; key themes will be discussed, recording and provided back to the community/participants for review.

This will provide a detailed thematic analysis of participant’s experience of their COPD assessment. Common themes and differences between participant sub-groups (age, sex, GOLD classification and place where CT scan undertaken) will also be described. nVIVO software will be used to will be code, categorise and manage the data from the interview transcripts.

Note: an unpublished NZ study that undertook qualitative interviews with COPD patients found that many, especially those with mild and moderate disease, were not aware that their symptoms were abnormal because of the slow onset and gradual decline of their respiratory function and consequently did not raise these as issues with their health professionals.

Analysis of data collected during the recruitment of participants and study data collection will be used to answer the following quantitative questions around data:

- Provide an early description of LCS outcomes (including number of cancers detected, stage of cancers etc.). In addition to providing a description, these data will be used to update the cost effectiveness model developed previously by our research group^45^.
- What is the accuracy of recording of smoking history in general practice?
- What % of Māori ‘not never smokers’ aged 55-74 in a general practice are potentially eligible for lung cancer screening
- What % of potentially eligible people agree to undergo risk assessment
- What % of those undergoing risk assessment are eligible for lung cancer screening
- What % of those eligible for lung cancer screening decide to proceed with screening
- What % of those who decide to proceed with screening attend their appointment for screening.
- What % of patients with ctDNA identified had a nodule identified on CT scan?
- What is the concordance between patient-reported smoking history and blood plasma estimation of smoking exposure?

Table 3: Implementation science dimensions and outcomes

| **Dimension** | **Element/level** | **Outcomes** |
| --- | --- | --- |
| Reach | Describe target audience & proportion reached | Participation proportion for Primary outcome 1 - of proportion of eligible population completed risk assessment  Number excluded and reasons  Record contact type responded to (letter, phone, text, social) |
|  | Comparison of sample to the target population | Representativeness of risk assessment participants and non-participants (by age, gender and smoking history)  Representativeness of CT scan participants compared to those eligible for CT scan but did not consent to one/attend for scan |
| Effectiveness | CT scan uptake | Participation proportion for Primary outcome 2 - proportion of people eligible and who complete CT scan, by invitation arm  Number excluded and reasons |
|  | Quality of life  Participant experience | We will include a dimension based (Hua Oranga) (see Appendix 13)  Participant interview including anxiety and appropriateness of SDM, level of information, study materials and value of screen to participant and whānau |
| Adoption | Staff | Number and description of delivery agents (e.g. practice nurse, GP) per practice. Number and description of non-participants.  Number of staff attending standardised training session including an implementation manual and guide to the advanced form data entry system embedded in their Practice Management System (PMS). Pre and post training survey of knowledge and confidence, particularly in providing SDM. Survey include ability to feedback to improve future training (see Appendix 17)  For all above measures, timelines will also be monitored. |
|  | Setting | Proportion of primary care practices offered participation who choose to participate  Characteristics of participating and non-participating practices |
|  | System impacts | Audit against readiness assessment assumptions |
| Implementation | Setting | Number of claims & cost for completed screen events per practice  Triangulated with completion of the advanced form (checklist tool) for screen event – this tool will capture the key events for fidelity, time to complete the screen, text field for adaptations/issues/fixes to consider  Fidelity triangulated with research nurse observations of risk assessment completion  Ongoing partnership with providers and key stakeholders with iterative feedback including on barriers and adaptations made  Updating our cost effectiveness model with trial data |
| Maintenance | Individuals | Incorporation of Consumer Advisory Group feedback ongoing  Participant and whānau questionnaires including asking whether they would recommend participation to friends & whānau, what more could be done to reach eligible people, recommendations (covers a range of RE-AIM dimensions)  Although not within this protocol, we are planning for a (funding dependent) 2^nd^ and 3rd screening round. We propose to contact round 1 participants at 12 months post intervention to determine willingness to be re-screened |
|  | Setting | Debrief post trial with key stakeholders including consumer advisory group, primary care and hospital stakeholders.  Provider focus groups and/or interviews (covers a range of RE-AIM dimensions) |

### Smoking cessation advice

All participants who are current smokers will be provided with brief advice to quit, offered nicotine replacement or other pharmacological treatments to support smoking cessation, and offered referral to quit services. For participants whose calculated risk means they are not eligible for lung cancer screening this will be provided at the completion of the risk assessment process. Participants who are eligible for screening but choose not to proceed with a CT scan will receive this (brief advice to quit, offers of medications and referral to cessation services) at that decision point. Participants who proceed to screening will be offered this when they are given their results. All relevant staff will receive training in smoking cessation support, and DHB and PHO smokefree services are aware of the project and are supportive.

## Statistical Consideration and Data Analysis

### Sample size and statistical analysis

The sample size for the overall randomised trial is calculated based on the power required to test the hypothesised difference in the number of participants completing lung cancer screening following an invitation to the study. Our sample size will provide more than sufficient power to test the hypothesised difference in completion of risk assessment. Sample size was calculated using the formula cited in Hade et al^51^. Table 4 outlines the assumptions made in the sample size calculation.

Table 4: Assumptions used in power calculation (MAIN STUDY)

| **Parameter** | **Assumed value** | **Justification** |
| --- | --- | --- |
| Proportion of Māori aged 55-74 who meet eligibility criteria to be invited for screening | 24% | Smoking history data collected in our pilot of AAA screening (28%), reduced by 4% to allow for those excluded on other grounds (terminal illness etc.). |
| Intraclass correlation coefficient | 0.03 | Approximately the median value of those cited in Hade et al. |
| Average number of eligible patients per practice | 24 | From practice population registers. Practices with <10 expected eligible patients will be excluded. |
| Proportion of Māori in eligible age range recorded as not “never smokers” | 70% | Data from one of the Auckland PHOs. |
| Proportion of not never-smokers eligible who complete risk assessment in the primary care group | 80% | It is difficult to estimate this parameter as there is no similar experience to base it on. However it would seem reasonable that it is possible to obtain smoking history on 70% of the eligible practice population in the control group, and that this could be 10% higher in the intervention group where primary care may have a significant advantage. |
| Proportion of not never-smokers eligible who complete risk assessment in central hub group | 70% |  |
| Screening uptake in the central hub group | 40% | Based on uptake rates routinely achieved for Māori in other screening programmes including bowel screening. |
| Screening uptake rate in primary care group | 50% | A goal of 10% higher uptake rate than the control group is both achievable and clinically meaningful |
| Variance reduction from regression adjustment of individual-level outcomes. | 10% | Consistent with variance reduction assumptions used by Hade et al. |
| Variance reduction from regression adjustment of group-level outcomes. | 20% | Consistent with variance reduction assumptions used by Hade et al. |
| Statistical power to detect 10% increase in screening uptake | 80% | Standard statistical power selection |
| Acceptable Type I error rate | 5% | Standard statistical significance selection |

Due to a lack of pilot studies from which to estimate the statistical power of the plasma biomarker analyses to be used here, plasma biomarkers were not included in these sample size calculations. This study will provide pilot data to inform power calculations for future studies.

#### COPD assessment

International studies have found high acceptance of spirometry within LCS programmes (>90%)^39,40^. We conservatively estimate that 400 (80%) of our LCS participants will consent to COPD assessment. Of these, we assume at least 200 (50%) will meet the criteria for a COPD diagnosis, for whom we can assess changes in baseline management after sending the report to the GP. Some improvement in COPD management over time may be expected following a spirometry test simply due to (normal) ongoing management by their GP or by the Hawthorne effect from being included in the study and having a spirometry test. We expect this to seen in about 10% of patients and if the proportion with improvements is significantly greater than 10%, then this might be attributable to the effect of the reporting and management recommendations that we will be providing to the GP. With a sample of 200 the study has 85% power to detect that a measured improvement in management is significantly greater than 10% when the actual proportion is 17% or more (95% significance level). Descriptive analysis of quantitative data will include frequency distributions (categorical and ordinal data); mean values for normally distributed numerical data; and median, minimum and maximum values and interquartile ranges for non-normally distributed numerical data. Comparison across subgroups of interest (e.g. GOLD classification, age, gender) will involve chi-square analysis for categorical variables; t-tests or analysis of variance for normally distributed or log transformed continuous variables; and the appropriate statistical test (e.g. Wilcoxin rank sums scores and Kruskal-Wallis tests) for non-normally distributed numerical data. GP’s COPD management variables (e.g. medications prescribed) at baseline and 4 months after sending repots to GPs will be categorised according to their consistency with COPD-X management recommendations for participant’s GOLD classification (Yes-fully, yes-partially and no). Each participant will be classified as having had improved management if the assessment changes from either: no to yes-fully or yes-partially; or from yes-partially to yes-fully. Wilson confidence limits will be calculated on this proportion and it will be tested for significance (one sample proportion test) versus the expected improvement proportion of 10%.

## Data management

PLCO_M2012_ risk and COPD assessment data will be entered into a RedCap database via a web-based interface. Other data collected during the invitation, risk assessment, SDM process, COPD assessment, biomarker analysis and qualitative evaluation of participant experiences will be entered into an electronic database at the time of collection and will be hosted in New Zealand at Waitematā District Health Board under university level firewall server protection. Data obtained from the screening CT scan e.g. scan result (normal, nodules, suspicious lesions, incidental findings), required follow up (none, nodule management, referral for investigation and management of suspicious and incidental findings), whether the management and follow up has occurred in a specified timeframe, and the results of the follow up will be stored on the study database. The databases will be housed at Waitematā DHB. All databases and electronic storage files will be stored securely and password protected. Data will be de-identified by use of a study ID for each participant. Access to health information will be restricted to key study personnel. A mechanism will be in place for data re-identification so that participants can be contacted should the need for follow up occur. Consent forms will be stored separately from study data.

### Data sharing and governance

No patient-level data will be shared outside of the research team for this study. A Māori data sovereignty assessment was conducted [54-55], confirming the requirement for Māori data governance. All data in this study will be Māori data and will be governed by the Māori members of the steering group, led by the Principal Investigator, with appropriate protections, data access agreements and management procedures consistent with Te Mana Rauranga (the Māori Data Sovereignty Network) principles

### Data and safety monitoring

Data will be monitored daily by the research nurse and research coordinator.

A person independent to the trial will be appointed to monitor the trial conduct. The trial will be monitored after 200 participants have been enrolled, at trial close-out and twice during the trial.

## Ethical considerations

### Informed consent

Informed consent will be sought from all practices and participants involved in the trial. PHOs will provide agreement and clinical approval to join the study. Practices will choose to participate in the study and agree to conduct the study procedures including training, result management and risk assessment (for primary care arm). Consent for participation will be taken by a suitably trained clinician or researcher who is familiar with the risks and benefits of the process.

Consent from participants will use a two-step process; verbal consent to participate in the trial will be sought from people on the list of people who are potentially eligible for the trial and for risk assessment. Eligible participants who decide to undergo a CT scan will be asked to provide written consent for the scan prior to undergoing it. Those who undergo COPD assessment will provide separate written consent. At the same time, separate consent will be sought for a member of the research team to contact the person’s GP to collect data about what actions the GP clinic has taken after receiving the result (patient notification), and to collect data about follow up CT scans, and diagnostic procedures done following the screening CT scan (GP and hospital records).

For the COPD sub-study, explicit consent will be sought to access data collected as part of the main LCS study that are relevant for this study: demographic data (age, gender, ethnicity), co-morbid conditions and smoking data (current smoking status, smoking history including duration and intensity of smoking). Explicit consent will also be sought to access both primary care records and hospital records for the following information: Documentation of COPD and COPD-related diagnoses (emphysema, asthma, chronic bronchitis, bronchiectasis); previous spirometry results; primary care management of COPD including non-pharmacological management and prescribing of medications for COPD and/or related diagnoses (short and long acting anti-muscarinic agents, short and long acting beta agonists, inhaled corticosteroids, oral glucocorticoids, and antibiotics for respiratory symptoms) in the previous 12 months; and the number of acute exacerbation of COPD, GP consults for respiratory symptoms, ED visits or hospitalisations for COPD/respiratory conditions in the last 12 months.

### Other ethical issues

- Possible distress for potentially eligible participants who do not meet risk threshold.
- Possible distress due to discussion of lung cancer or COPD.
- Risk of stigmatisation of smokers and ex-smokers – all people who interact with participants and whānau will be trained in mana-enhancing and mindful engagement to reduce stigmatisation.
- Clear processes needed for managing distress and training for all research team members.

### Adverse events and risks

There is a potential but unlikely risk of a participant suffering an adverse event as a result of participation in this study.

From overseas studies, we anticipate a 1.7% false positive rate when analysing scan results, which means that we anticipate that between 9 and 10 participants will need repeat scans and potentially other more invasive tests before they are given a result of no lung cancer detected. In addition, we anticipate approximately 108 participants that do not have lung cancer will nevertheless have an “indeterminate” scan and require repeat scanning or follow up.

Participants that are referred for further follow up as a result of their screening CT scan will be referred into the standard DHB treatment pathway and will be the responsibility of the treating secondary care physician. We will continue to collect data on outcomes for these participants as part of the study.

De-identified information on all participants referred for follow up will be shared with the study Steering Group. Other potential adverse events and risks of the study are listed below:

**False sense of security:** People who do not meet the risk threshold (so do not receive a scan) and those who have normal scans may incorrectly believe they are not at future risk of lung cancer. Participants will be made aware that being low risk or having a normal CT scan does not exclude future cancers and will be given information about lung cancer symptoms and what actions to take if they develop these symptoms in the future. They may be re-invited to screening in a future screening programme or research follow up study. There is also a small risk of a false negative result. All participants will be advised to see a doctor if they have symptoms of lung cancer in the future, even if their screen is negative.

Exposure to radiation: The radiation from low-dose CT scans is about 14 times that of an x-ray, and delivers about half of the radiation exposure that an average person would usually receive in a year. Having more than one CT scan may slightly increase a participant’s risk of cancer, however, at the relatively low doses of radiation used, the risk of developing cancer from a CT scan is so small that it is difficult to measure it. Participants will be made aware of this small risk

Further testing: If the CT scan identified nodules in the lungs, participants will be offered further tests. The investigation of positive screening scans using diagnostic assessment such as bronchoscopy may be associated with physical harm to participants. In addition, an abnormal result from a CT scan may cause anxiety whilst participants go through this process.

Over-diagnosis: Some small cancers may never have become a problem had they not been diagnosed through screening. Although the extent to which lung cancer LC is over-diagnosed by CT screening is contentious and currently unknown at an individual level, participants will be made aware of this risk.

Information about all these potential risks, and the potential benefits of participating in the study will be provided to participants during the informed consent and shared decision making processes. If whānau do not participate in invitation, risk assessment and shared decision making processes, participants will, if they wish to, be supported to consult with whānau and/or their healthcare providers prior to coming to a decision.

Confirming if follow-up is appropriate may involve a referral to a suitable health professional or specialist. Suitable counselling (clinical, genetic or emotional) may be necessary for participants, depending on the information uncovered. This will be assessed on a case by case basis by the study respiratory physician.

Ethical approval will be sought from the NZ HDEC ethics committee, and any substantial amendments or protocol deviations will be submitted to the approving committee for review.

## Outcomes and Significance

### Primary outcome

To determine the effectiveness of two invitation strategies: primary care vs central hub.

### Secondary outcomes

Secondary outcomes include:

**Eligibility for risk assessment:** Proportion identified as potentially eligible by PHO list who were eligible for risk assessment.

**Completion of Risk assessment:** Proportion of participants who were eligible for risk assessment that completed the risk assessment process. Also an assessment of the differences between the primary care and central hub groups.

**CT scan eligibility:** Proportion of those who underwent risk assessment that met risk threshold for a CT scan by PLCO_M2012_ and criteria-based eligibility.

**Effectiveness of engagement in shared decision making:** proportion of eligible participants that decide to proceed with screening after participating in a shared decision making process to discuss the risks and benefits.

**LCS screening uptake among those eligible:** proportion of those that agree to lung cancer screening who attended and underwent the scan.

- Participant and provider experience of LCS
- Experience and acceptability of the shared decision making and results management processes
- Participant burden using the tools outlined in the study methods
- Provider burden using the processes outlined in the GP clinic based arm of the study
  - The burden on staff and participants will be measured through follow-up interviews and/or focus groups conducted by a member of the research team following the implementation of the pilot.
- Provision of a description of LCS outcomes. This will include The positivity rate of CT scans; the proportion of scans with incidental findings; proportions who proceed to nodule management (interval scans); diagnostic investigations; incidental finding management; the number, stage and types of cancers; response to brief advice to quit; and smoking cessation medications provided and referrals made.
- Assessment of the accuracy of recording of smoking history in general practice

**Prevalence of COPD among those eligible for LCS:** proportion of those eligible who have a spirometry-based COPD diagnosis.

**Change in GP management following provision of results**

**COPD assessment process:**

- Participant and whānau experience of COPD assessment
  - Positive and negative experiences
  - Barriers and facilitators to participation
- Impact of being told the results of assessment
- Participant and whānau views on whether COPD assessment should be included in a future LCS programme, including suggestions for changes to the COPD assessment process.

**Biomarker study**

- Participant and whānau experience of biomarker assessment
  - Positive and negative experiences
  - Barriers and facilitators to participation
- Impact of being told the results of assessment (if applicable)
- Participant and whānau views on whether biomarker assessment should be included in a future LCS programme, including suggestions for changes to the process.

## References

- - - 1. Global Initiative for Chronic Obstructive Lung Disease. Global Strategy for the Diagnosis, Management, and Prevention of Chronic Obstructive Lung Disease. 2020 Report. USA: Global Initiative for Chronic Obstructive Lung Disease, 2020.
      2. BPAC NZ. Diagnosis and management of COPD in Māori and Pacific peoples. Best Practice Journal 2012;43:14-25.
      3. Rossi A, Butorac-Petanjek B, Chilosi M, et al. Chronic obstructive pulmonary disease with mild airflow limitation: current knowledge and proposal for future research – a consensus document from six scientific societies. International Journal of COPD 2017;12:2593-610.
      4. Ministry of Health. New cancer registrations 2017 [Internet]. Wellington: Ministry of Health; 2019 [updated 2020 Sept 30; cited 2020 nov 20]. Available from: <https://www.health.govt.nz/publication/new-cancer-registrations-2017>
      5. Ministry of Health. Cancer: Historical summary 1948-2017 [Internet]. Wellington: Ministry of Health; 2019 [cited 2020 Nov 20]. Available from: <https://www.health.govt.nz/publication/cancer-historical-summary-1948-2017>
      6. Ministry of Health. Tatau Kahukura: Māori Health Chart Book 2015 [Internet]. 3rd edition. Wellington: Ministry of Health; 2015 [cited 2020 Nov 20]. Available from: <https://www.health.govt.nz/publication/tatau-kahukura-maori-health-chart-book-2015-3rd-edition>
      7. Teng AM, Atkinson J, Disney G, Wilson N, Sarfati D, McLeod M, et al. Ethnic inequalities in cancer incidence and mortality: Census-linked cohort studies with 87 million years of person-time follow-up. BMC Cancer [Internet]. 2016 [cited 2020 Nov 20]; 16(1). Available from: <https://link.springer.com/article/10.1186/s12885-016-2781-4>
      8. Ministry of Health. New Zealand Cancer Action Plan 2019-2029 – Te Mahere mō te Mate Pukupuku o Aotearoa 2019-2029 [Internet]. Wellington (NZ): Ministry of Health; 2019 [cited 2020 Nov 13]. Available from: https://www.health.govt.nz/publication/new-zealand-cancer-action-plan-2019-2029
      9. De Koning H, et al. Effects of volume CT lung cancer screening: mortality results of the NELSON randomised-controlled population based trial. Journal of Thoracic Oncology 2018;13(10)
      10. Aberle DR, et al. Reduced lung-cancer mortality with low-dose computed tomographic screening. N Engl J Med 2011;365(5):395-409. doi: 10.1056/NEJMoa1102873
      11. Huang K-L, et al. Effects of low-dose computed tomography on lung cancer screening: a systematic review, meta-analysis, and trial sequential analysis. BMC Pulmonary Medicine 2019;19(1):126.
      12. van der Aalst CM, Ten Haaf K, de Koning HJ. Implementation of lung cancer screening: what are the main issues? Transl Lung Cancer Res. 2021;10(2):1050-1063
      13. Rivera MP, Katki HA, Tanner NT, et al. Addressing Disparities in Lung Cancer Screening Eligibility and Healthcare Access. An Official American Thoracic Society Statement. Am J Respir Crit Care Med. 2020;202(7):e95-e112
      14. Rankin N, McWilliams A, Marshall H. Lung cancer screening implementation: Complexities and priorities. Respirology 2020 25(S2) 5-23
      15. Kennedy MPT, Cheyne L, Darby M, et al. Lung cancer stage-shift following a symptom awareness campaign. Thorax 2018;73:1128-1136.
      16. Tammemagi MC, ten Haag K, Toumazis I, Kong CY, Han SS, Jeon J, et al. Development and validation of a multivariable lung cancer risk prediction model that includes low-dose computed tomography screening results: A secondary analysis of data from the National Lung Cancer Screening Trial. JAMA Netw Open [Internet]. 2019 [cited 2020 Nov 11]; 2(3): e190204. Available from: <https://jamanetwork.com/journals/jamanetworkopen/fullarticle/2726714#:~:text=The%20PLCOm2012%20is%20a%20lung,Germany%2C%20Australia%2C%20and%20Canada.&text=One%20appropriate%20PLCOm2012%20risk%20threshold,1.5%25%206%2Dyear%20risk>.
      17. Tammemagi MC. Selecting lung cancer screenees using risk prediction models – where do we go from here. Transl Lung Cancer Res [Internet]. 2018 [cited 2020 Nov 18]; 7(3): 243-253. Available from: <https://www.ncbi.nlm.nih.gov/pmc/articles/PMC6037970/>
      18. Haaf K, Jeon J, Tammemägi MC, et al. (2017) Risk prediction models for selection of lung cancer screening candidates: A retrospective validation study. PLoS Med 14(4): e1002277
      19. Tammemagi MC, Katki HA, Hocking WG, et al. Selection criteria for lung-cancer screening. The New England journal of medicine 2013;368(8):728-36
      20. González Maldonado S, Delorme S, Hüsing A, et al. Evaluation of Prediction Models for Identifying Malignancy in Pulmonary Nodules Detected via Low-Dose Computed Tomography. JAMA Netw Open. 2020;3(2):e1921221.
      21. Hopkins R, Kendall C, Gamble G, et al. Are New Zealand Māori more susceptible to smoking related lung cancer? - A comparative case-case study. EC Pulmonology and Respiratory Medicine 2019;8(1):72-91.
      22. Durham A, Adcock I. The relationship between COPD and lung cancer. Lung Cancer 2015;90(2):121-27.
      23. *Young R, Hopkins R. Chronic obstructive pulmonary disease (COPD) and lung cancer screening. Translational Lung Cancer Research 2018;7(3):347-60.
      24. Telfar-Bernard L, Zhang J. The Impact of Respiratory Disease in New Zealand: 2016 Update. Wellington, New Zealand: The Asthma Foundation, 2017.
      25. Ministry of Health. Tatau Kahukura: Māori Health Chart Book 2015 (3rd edition). In: Health Mo, ed. Wellington: Ministry of Health, 2015.
      26. Robson B, Harris R. Hauora: Māori Standards of Health IV. A study of the years 2000–2005. Wellington: Te Rōpū Rangahau Hauora a Eru Pōmare 2007.
      27. Telfar-Bernard L, Zhang J. The Impact of Respiratory Disease in New Zealand: 2018 Update. Wellington: The Asthma Foundation, 2019.
      28. Auckland District Health Board. Health Needs Assessment. Auckland: Auckland District Health Board, 2019.
      29. Waitemata District Health Board. Health Needs Assessment Auckland: Waitemata District Health Board, 2019.
      30. Heffler E, Crimi C, Mancuso S, et al. Misdiagnosis of asthma and COPD and underuse of spirometry in primary care unselected patients. Respir Med 2018;142:48-52.
      31. Rothnie KJ, Chandan JS, Goss HG, et al. Validity and interpretation of spirometric recordings to diagnose COPD in UK primary care. Int J Chron Obstruct Pulmon Dis 2017;12:1663-68.
      32. Nardini S, Annesi-Maesano I, Simoni M, et al. Accuracy of diagnosis of COPD and factors associated with misdiagnosis in primary care setting. E-DIAL (Early DIAgnosis of obstructive lung disease) study group. Respir Med 2018;143:61-66.
      33. Duvall K, Frank GW. Identifying chronic obstructive pulmonary disease in primary care of urban underserved patients: tools, applications, and challenges. J Natl Med Assoc 2010;102(7):570-8.
      34. Yawn B, Kim V. COPD in Primary Care: Key Considerations for Optimized Management: Treatment Options for Stable Chronic Obstructive Pulmonary Disease: Current Recommendations and Unmet Needs. J 2018;67(2 Suppl):S28-S37.
      35. Price D, West D, Brusselle G, et al. Management of COPD in the UK primary-care setting: an analysis of real-life prescribing patterns. International Journal of COPD 2014;9:889-905.
      36. Lung Foundation of Australia. COPD-X Concise Guide for Primary Care: Lung Foundation of Australia 2019.
      37. CAT website. The COPD Assessment Test 2019 [Available from: https://www.catestonline.org/hcp-homepage.html accessed 1 July 2019 2019.
      38. Kocks JWH, Blom CMG, Kasteleyn MJ, et al. Feasibility and applicability of the paper and electronic COPD assessment test (CAT) and the clinical COPD questionnaire (CCQ) in primary care: a clinimetric study. NPJ Prim Care Respir Med 2017;27(1):20.
      39. Ruparel M, Quaife S, Dickson J, et al. Prevalence, Symptom Burden, and Under diagnosis of Chronic Obstructive Pulmonary Disease in a Lung Cancer Screening Cohort. Annals of the American Thoracic Society 2020
      40. Balata H, Harvey J, Barber P, et al. Spirometry performed as part of the Manchester community-based lung cancer screening programme detects a high prevalence of airflow obstruction in individuals without a prior diagnosis of COPD. Thorax 2020; epublished:1-6.
      41. Blackmore C, Johnson-Warrington VL, Williams JEA, et al. Development of a training program to support health care professionals to deliver the SPACE for COPD self-management program. International Journal of Chronic Obstructive Pulmonary Disease 2017;12:1683.
      42. Vega-Sanchez AE, Tellez-Navarrete NA, Perez-Padilla R. Chronic Obstructive Pulmonary Disease: Perspectives for Primary Health Care. Rev Invest Clin 2019;71(1):55-63.
      43. Lung Foundation of Australia. The COPD-X Plan: Australian and New Zealand Guidelines for the management of Chronic Obstructive Pulmonary Disease 2019: Lung Foundation of Australia, 2019.
      44. Jaine R. et al. Cost-effectiveness of a low-dose computed tomography screening programme for lung cancer in New Zealand. Lung Cancer 124, (2018):233-40. <https://pubmed.ncbi.nlm.nih.gov/30268467/>
      45. McLeod M, Sandiford P, Kvizhinadze G, et al Impact of low-dose CT screening for lung cancer on ethnic health inequities in New Zealand: a cost-effectiveness analysis BMJ Open 10(2020). <https://bmjopen.bmj.com/content/10/9/e037145> doi:10.1001/jamanetworkopen.2019.21221
      46. Standardization of Spirometry 2019 Update: An Official American Thoracic Society and European Respiratory Society Technical Statement.
      47. Landes, S. J., McBain, S. A., & Curran, G. M. (2020). An introduction to effectiveness-implementation hybrid designs. Psychiatry research, 283, 112630.
      48. Glasgow, R. E., Vogt, T. M., & Boles, S. M. (1999). Evaluating the public health impact of health promotion interventions: the RE-AIM framework. American journal of public health, 89(9), 1322-1327.
      49. Oetzel J, Scott N, Hudson M, et al. Implementation framework for chronic disease intervention effectiveness in Māori and other indigenous communities. Globalization and Health 2017;13(1)
      50. Canadian Partnership against Cancer. Implementation Planning Guide for Programmatic Lung Cancer Screening (2020) (https://www.partnershipagainstcancer.ca/topics/lung-screening-resources/)
      51. Hade EM, Murray DM, Pennell ML, et al. Intraclass correlation estimates for cancer screening outcomes: estimates and applications in the design of group-randomized cancer screening studies. Journal of the National Cancer Institute Monographs 2010;2010(40):97
      52. <https://www.uspreventiveservicestaskforce.org/uspstf/recommendation/lung-cancer-screening>
      53. Phallen J, Sausen M, Adleff V, et al. Direct detection of early-stage cancers using circulating tumor DNA. Sci Transl Med. 2017 Aug 16;9(403): eaan2415 (<https://www.clinicaltrials.gov/ct2/show/NCT03934866>)
      54. Mathios D, Johansen JS, Cristiano S, et al. Detection and characterization of lung cancer using cell-free DNA fragmentomes. Nat Commun. 2021 Aug 20;12(1):5060
      55. Shen SY, Singhania R, Fehringer G, et al. Sensitive tumour detection and classification using plasma cell-free DNA methylomes. Nature 2018, 563:579-583
      56. Grunnet M, Sorensen JB. Carcinoembryonic antigen (CEA) as tumor marker in lung cancer. Lung Cancer. 2012;76(2):138–143
      57. Salgia R, Harpole D, Herndon JE 2nd, Pisick E, Elias A, Skarin AT. Role of serum tumor markers CA 125 and CEA in non-small cell lung cancer. Anticancer Res. 2001;21:1241–1246
      58. Ruochuan Zang, Yuan Li, Runsen Jin, et al. (2019) Enhancement of diagnostic performance in lung cancers by combining CEA and CA125 with autoantibodies detection, OncoImmunology, 8:10, DOI: [10.1080/2162402X.2019.1625689](https://doi.org/10.1080/2162402X.2019.1625689)
      59. Risberg B, Tsui DWY, Biggs H, et al: Effects of Collection and Processing Procedures on Plasma Circulating Cell-Free DNA from Cancer Patients. J Mol Diagn 2018, 20:883-892
      60. Tantoh, D.M., Lee, KJ., Nfor, O.N. et al. Methylation at cg05575921 of a smoking-related gene (AHRR) in non-smoking Taiwanese adults residing in areas with different PM2.5concentrations. Clin Epigenet 11, 69 (2019). <https://doi.org/10.1186/s13148-019-0662-9>
      61. Pidsley, R., Zotenko, E., Peters, T. J., et al. (2016). Critical evaluation of the Illumina MethylationEPIC beadchip microarray for whole-genome DNA methylation profiling. Genome Biol. 17:208
      62. Lasham A, Fitzgerald S, Knowlton N, et al. A predictor of early disease recurrence in breast cancer patients using a cell-free RNA and protein liquid biopsy. Clinical Breast Cancer, 2020, 20(2), 108-116
      63. New Zealand Medical Association NZ COPD Guidelines 2021

## Appendices

Appendix 1: Lung cancer screening study information booklet

Appendix 2: PIS Risk Assessment

Appendix 3: PIS CT Scan

Appendix 4: Aid for Shared Decision Making

Appendix 5: PIS for clinical staff for interviews / focus groups

Appendix 6: PLCOm2012 Risk Assessment Model

Appendix 7: Script for consent to Shared Decision Making

Appendix 8: Survey after a Risk Assessment – for participants found not to have a high risk of lung cancer (and therefore ineligible for a CT scan)

Appendix 9: Survey about Risk Assessment and Shared Decision Making (for those that decline a CT scan)

Appendix 10: Survey about Shared Decision Making and CT scan (for all participants having a CT scan)

Appendix 11: Survey after a CT scan (selected participants)

Appendix 12: Whānau Tautoko Survey

Appendix 13: Hua Oranga Questionnaire

Appendix 14: Survey after COPD Assessment (all COPD participants)

Appendix 15: Qualitative interview after COPD assessment

Appendix 16: Survey after Biomarker Assessment (all Biomarker participants)

Appendix 17: Pre and post training evaluation form

### Appendix 1: Lung cancer screening study information booklet

###
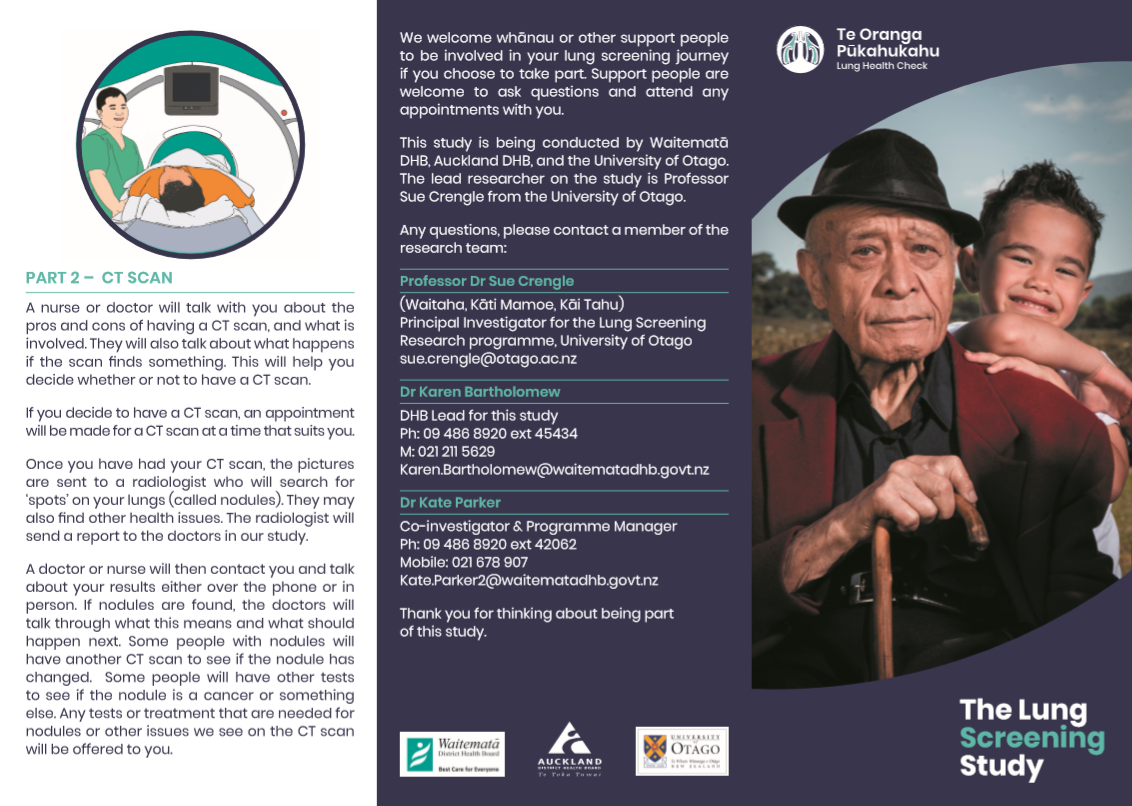


###
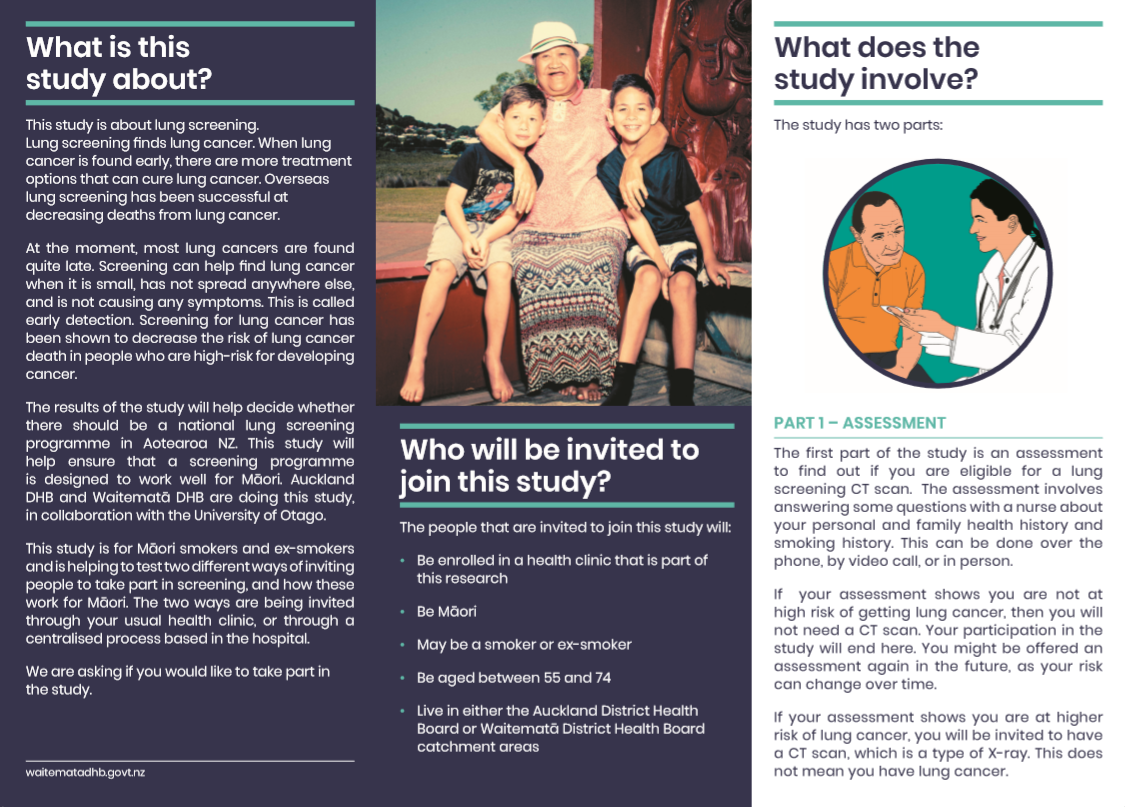


### Appendix 2: PIS Risk Assessment

See additional attachment

### Appendix 3: PIS CT Scan

See additional attachment

### Appendix 4: Aid for Shared Decision Making

See additional attachment

### Appendix 5: PIS for clinical staff for interviews / focus groups

See additional attachment

### Appendix 6: PLCO_m2012_ Risk Assessment Model

The PLCOm^2012^ risk assessment model will be used to determine risk level for participants that consent to undergo risk assessment.

The PLCO_m2012_ risk assessment model will collect the following information to assess a participants’ risk of developing lung cancer over the next 6 years:

- Age in years
- Education level (less than high school graduate, high school graduate, post high school training, some college, college graduate, postgraduate / professional)
- Body Mass Index (BMI) (weight in kg/height in m^2)
- Does the participant have COPD, emphysema or bronchitis?
- Personal history of cancer
- Family history of lung cancer
- Ethnicity (Māori)
- Smoking status (former smoker or current smoker)
- Average number of cigarettes smoked per day
- Duration smoked
- Years since quitting smoking (if applicable)

In addition to the model parameter, we will also ask participants whether they have worked in jobs for 5 years or more where they have been regularly exposed to any of the following;

-          Asbestos ( e.g. construction, electrical, demolition)?

-          Silica ( e.g. construction, concrete cutting, drilling, working with artificial stone)?

-          Welding fume ( operating arc, stick, MiG, tig, gas as routine part of job)?

-          Have you worked in construction for longer than 5 years in any role?

Furthermore, we also wish to collect information on a person’s passive smoking history. They will be asked the following questions:

The following questions are about your exposure to other people’s smoke, often called “secondhand smoke”

As a child and youth under the age of 18 years, was your exposure to secondhand smoke at home:

🞏 minimal or zero 🞏 Mild 🞏 Moderate 🞏 Heavy

As an adult, was your exposure to secondhand smoke at home:

🞏 minimal or zero 🞏 Mild 🞏 Moderate 🞏 Heavy

As an adult, was your exposure to secondhand at work:

🞏 minimal or zero 🞏 Mild 🞏 Moderate 🞏 Heavy

As an adult, was your exposure to secondhand smoke at private functions: (eg. parties, wedding, meetings,private clubs)

🞏 minimal or zero 🞏 Mild 🞏 Moderate 🞏 Heavy

As an adult, was your exposure to secondhand smoke in public places: (eg. buses, bus stops, bingo halls, bars, clubs)

🞏 minimal or zero 🞏 Mild 🞏 Moderate 🞏 Heavy

### Appendix 7: Script for consent to Shared Decision Making

Te Oranga Pūkahukahu Lung Screening
Telephone script to consent to SDM regarding having a CT scan

Kia ora [NAME]. Its [staff members name] from the lung cancer screening study. I am calling to talk to you about whether you would like to know more about participating in the next stage of the lung cancer screening study.

Your risk assessment results showed that you are eligible for a lung screening test (using a CT scan).

Now you can make a decision about whether you want to have a CT scan. Your whānau can be part of making this decision too if you would like them to be.

We sent you a booklet called [XX] that is about the CT scan and what happens after you have the scan. Have you got that booklet yet?

We would like to talk you through this information to help you decide if having a CT scan is right for you. This discussion can be done either in-person or over the phone. Your whānau or other support people can join you if you want them to.

Would you like to arrange a time for us to go through this information with you? *[Agree a time to continue the discussion – can do it at the time of the call if that is convenient for the participant]*

Whether or not you decide to go ahead with having a lung screening CT scan, we would like to keep a written record of your answers during this decision making process. This will help us to make the process better in the future.

If you decide to go ahead with having a lung screening CT scan we will book an appointment for a CT scan at a time that suits you. When you go for the scan you will be asked to sign a consent form for the scan.

**Verbal record of agreement to keep a record of SDM discussion**

I agree that the information collected about me during this discussion and decision making process will be included in the study.

Declaration by participant:

I consent to take part in this study *[recorded by researcher]*

Name: Date:

**Declaration by member of research team:**

I have given a verbal explanation of the research project to the participant, and have answered the participant’s questions about it.

I believe that the participant understands the study and has given verbal informed consent to participate.

Researcher’s name:

Signature: Date:

### Appendix 8: Survey after a Risk Assessment – for participants found not to have a high risk of lung cancer (and therefore ineligible for a CT scan)

“Survey about your experiences of the lung cancer screening study”

Tēnā koe

As part of our study we are checking your experiences of the Assessment part of the study. Please complete this survey to help us improve the way lung screening is offered in the future. This survey is voluntary.

| 1. Was the study explained to you before your risk assessment? | Yes 🞏 No 🞏 |
| --- | --- |
| 1. Was the information given to you about the study informative and helpful? | Yes 🞏 No 🞏 |
| 1. Was the risk assessment clearly explained to you before it started? |  |
| 1. What would have improved the information you received? (E.g. pictures or diagrams, less jargon) |  |
| 1. What was your first reaction when you were invited to take part in the study? (for example, were you curious/ worried / confused / excited?) |  |
| 1. How did you feel when the Assessment showed you had a normal risk of lung cancer and did not need more screening? |  |
| 1. Do you have any other comments on what worked well or could be improved about this process? |  |
| 1. Were you offered any health related information such as information on smoking?   If not, would you like to be referred to some of our services? | Yes 🞏 No 🞏  Yes 🞏 No 🞏 |

### Appendix 9: Survey about Risk Assessment and Shared Decision Making (for those that decline a CT scan)

“Survey about your experiences of the lung cancer screening study”

Tēnā koe

As part of our study we are checking your experiences of the lung cancer screening study. Please complete this survey to help us improve the way lung screening is offered in the future. This survey is voluntary.

| 1. Was the study explained to you before your risk assessment? | Yes 🞏 No 🞏 |
| --- | --- |
| 1. Was the information given to you about the study informative and helpful? | Yes 🞏 No 🞏 |
| 1. Was the risk assessment clearly explained to you before it started? |  |
| 1. What would have improved the information you received? (E.g. pictures or diagrams, less jargon) |  |
| 1. What was your first reaction when you were invited to take part in the study? (for example, were you curious/ worried / confused / excited?) |  |
| 1. How did you feel when the Assessment showed you had a higher risk of lung cancer and were eligible for a CT scan? |  |
| 1. Were you offered any health related information such as information on smoking?   If not, would you like to be referred to some of our services? | Yes 🞏 No 🞏  Yes 🞏 No 🞏 |
| 1. Do you remember the study nurse explaining the risks and benefits of lung cancer screening to you when you were deciding whether to have a CT scan or not? | Yes 🞏 No 🞏 |
| 1. Did you have this discussion in person/over the phone/ over video call? | In person 🞏 By phone 🞏  By video 🞏 |
| 1. If over the phone or video call, did you find this method as effective as an in-person appointment? | Yes 🞏 No 🞏 |
| 1. Did this discussion help you to make your decision to take part in lung screening? | Yes 🞏 No 🞏 |
| 1. Did you feel comfortable talking with the nurse or doctor? | Yes 🞏 No 🞏 |
| 1. Did you feel you were fully informed of the risks and benefits of lung cancer screening? | Yes 🞏 No 🞏 |
| 1. How did you feel after you made your decision about the CT scan? |  |
| 1. What went well during your discussion with the nurse when you were making a decision about whether or not to have a CT scan? |  |
| 1. Was there anything during your discussions that didn’t go well? |  |
| 1. What can we do to make the decision making process better? |  |
| 1. Do you have any other comments on what worked well or could be improved about this process? |  |

### Appendix 10: Survey about Shared Decision Making and CT scan (for all participants having a CT scan)

“Survey about your experiences of the lung cancer screening study”

Tēnā koe

Thank you for taking part in Te Oranga Pūkahukahu Lung Health Check. We have a few questions about your recent experiences with us, which we hope will improve our service.

| 1. Was the study explained to you before your risk assessment? | Yes 🞏 No 🞏 |
| --- | --- |
| 1. Was the information given to you about the study informative and helpful? | Yes 🞏 No 🞏 |
| 1. Was the risk assessment clearly explained to you before it started? |  |
| 1. What would have improved the information you received? (E.g. pictures or diagrams, less jargon) |  |
| 1. What was your first reaction when you were invited to take part in the study? (for example, were you curious/ worried / confused / excited?) |  |
| 1. How did you feel when the Assessment showed you had a higher risk of lung cancer and were eligible for a CT scan? |  |
| 1. Were you offered any health related information such as information on smoking?   If not, would you like to be referred to some of our services? | Yes 🞏 No 🞏  Yes 🞏 No 🞏 |
| 1. Do you remember the study nurse explaining the risks and benefits of lung cancer screening to you when you were deciding whether to have a CT scan or not? | Yes 🞏 No 🞏 |
| 1. Did you have this discussion in person/over the phone/ over video call? | In person 🞏 By phone 🞏  By video 🞏 |
| 1. If over the phone or video call, did you find this method as effective as an in-person appointment? | Yes 🞏 No 🞏 |
| 1. Did this discussion help you to make your decision to take part in lung screening? | Yes 🞏 No 🞏 |
| 1. Did you feel comfortable talking with the nurse or doctor? | Yes 🞏 No 🞏 |
| 1. Did you feel you were fully informed of the risks and benefits of lung cancer screening? | Yes 🞏 No 🞏 |
| 1. How did you feel after you made your decision about the CT scan? |  |
| 1. What went well during your discussion with the nurse when you were making a decision about whether or not to have a CT scan? |  |
| 1. Was there anything during your discussions that didn’t go well? |  |
| 1. What can we do to make the decision making process better? |  |

Having a CT scan

1. How was your experience of getting a CT scan?

|  |  |  |  |  |
| --- | --- | --- | --- | --- |
| I was very happy with the way it went | It was OK | Neutral /  Don’t know | It wasn’t great | I was unhappy with the way it went |

Please add any comments you would like to make:

1. Would you recommend having a CT scan for lung screening to other people? Yes/No
   Please can you explain your answer:
2. Did the information you were given prepare you what happened during the CT scan?

|  |  |  |  |  |
| --- | --- | --- | --- | --- |
| Yes I was very prepared | Prepared | Neutral /  Don’t know | Slightly unprepared | No I was very unprepared |

Please add any comments you would like to make:

1. What can we do to make the CT scan process better?

Please give us your suggestions for improvements:

1. Is there anything else you want to say?:

### Appendix 11: Survey after a CT scan (selected participants)

Tēnā koe

As part of our study we are checking your experience of the CT scan for lung screening. Please complete this survey to help us improve the way lung screening is offered in the future. This survey is voluntary.

| 1. Are you happy with the information given to you to prepare for the CT scan? | Yes 🞏 No 🞏 |
| --- | --- |
| 1. Did you feel any worry or anxiety about having a CT scan? | Yes 🞏 No 🞏 |
| 1. If yes, what was the cause of your worry or anxiety? | I was afraid of the results 🞏  I was uncomfortable having a CT scan 🞏  I tend to be worried or anxious about medical tests 🞏  Other 🞏  Please comment: |
| 1. Did the study staff help you with managing your worry or anxiety? | Yes 🞏 No 🞏 |
| 1. Are you satisfied with the quality of care you received? | Yes 🞏 No 🞏 |
| 1. How was your experience of having a CT scan? | Fine 🞏 Better than expected 🞏  Worse than expected 🞏 |
| 1. Did you feel safe going into the CT scan? | Yes 🞏 No 🞏 |
| 1. Did anything happen during the CT scan that would put you off consenting to a CT scan in the future? | Please comment: |
| 1. Were you told when you would get your CT scan results? | Yes 🞏 No 🞏 |
| 1. Did you get your CT scan results on time? | Yes 🞏 No 🞏 |
| 1. Is there anything that could have prevented you from attending the CT scan? (such as travel costs, family commitments etc.) | Please comment: |
| 1. Did you get enough information about your CT scan results? | Yes 🞏 No 🞏 |
| 1. Was the explanation of your CT scan result easy to understand? | Yes 🞏 No 🞏 |
| 1. Do you know what your next steps are? (E.g. doctors appointment, follow up scan in one year etc.)? | Yes 🞏 No 🞏 |
| 1. Would you do lung cancer screening again in the future if it was available ? | Yes 🞏 No 🞏 |
| 1. Have you made any health changes since taking part in this study? | Yes 🞏 No 🞏 |
| 1. Would you recommend lung cancer screening to your whānau and friends? | Yes 🞏 No 🞏 |

### Appendix 12: Whānau Tautoko Survey

Tēnā koe,

Thank you for agreeing to take part in this survey and for supporting your whānau member through their lung screening journey.

This survey is for whānau tautoko (support people) to share your whakaaro (thoughts) to support the kaupapa of the lung screening study.

By answering these questions, you are consenting to the research team including this information in an analysis of how well the lung screening research programme is working. All answers are anonymous and you will not be able to be identified.

You do not have to participate in this questionnaire if you don’t want to, and if you say no, this will in no way affect the care your whānau member receives.

Please make sure you are completing the correct survey: this is for **whānau tautoko**.

1. Was your whānau member treated with respect during the study?
2. What can we do to improve the way we support people who are going through lung screening*?*
3. What can we do to help whānau who are supporting someone going through lung screening?
4. Was the information we gave to your whānau member, and to the whānau, too much / just right / not enough?
5. Was the assessment process easy to understand?
6. Was the shared decision making process easy to understand?
7. Did the system to book the appointment work well for your whānau?
8. Did the appointment times that were offered to your whānau work? If no, why not?
9. Is there anything you would like us to know about your whānau experience with lung cancer screening?
10. What is your gender? Male Female Gender diverse Prefer not to answer
11. What age are you? 16-19 | 20-29 | 30-39 | 40-49 | 50-59 | 60-69 | 70-79 | 80+
12. Do you identify as (please tick all that apply)
    New Zealand European|Māori | Samoan| Cook Islands Māori |Tongan |Niuean |Chinese| Indian| Other
13. If you identify as Māori, which iwi do you whakapapa/connect to? (Can be more than 1)
    Iwi:

### Appendix 13: Hua Oranga Questionnaire


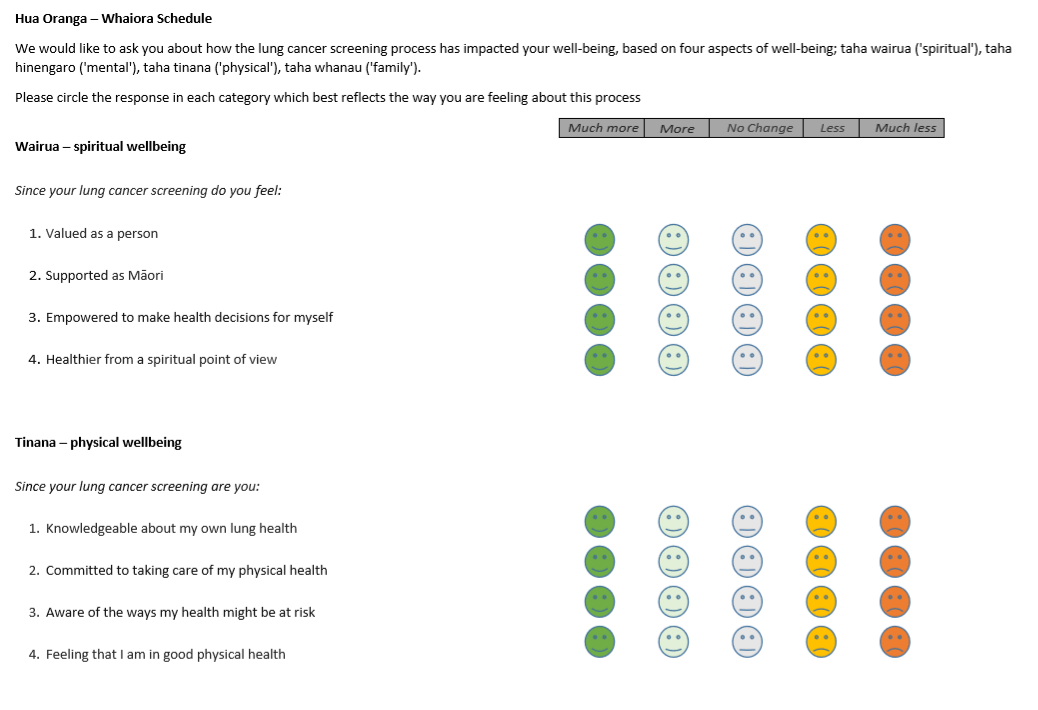


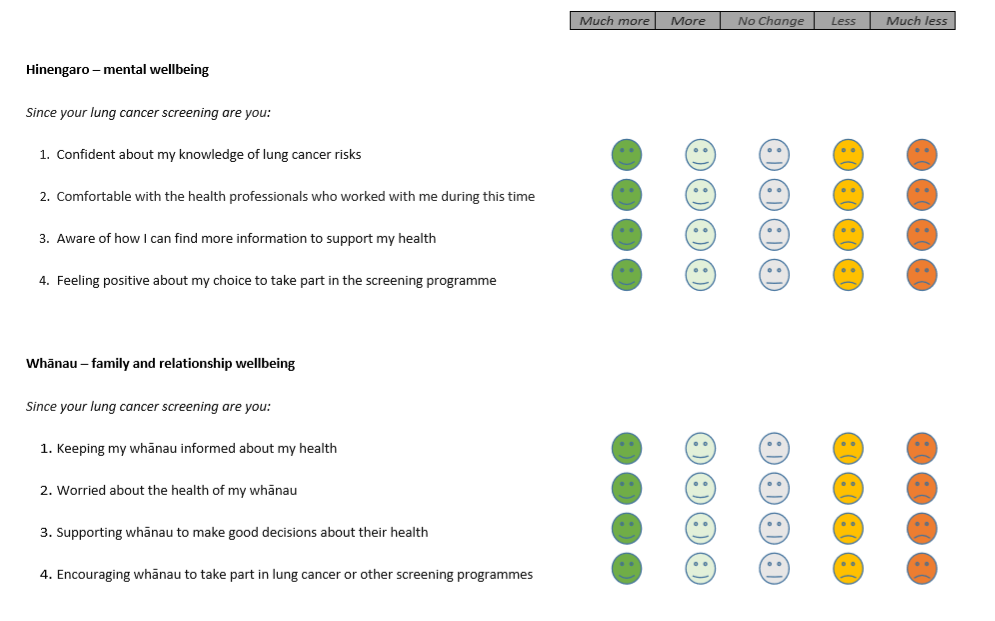


### Appendix 14: Survey after COPD Assessment (all COPD participants)

Tēnā koe

Thank you for taking part in an assessment for COPD. We would like to know how you found this. Please complete this survey – this will help us improve the way we do COPD assessments in the future. This survey is voluntary. (Note – this interview will be conducted with the study nurse, immediately after the assessment).

| 1. Was the COPD assessment explained to you in a way that you understood? | Yes 🞏 No 🞏 |
| --- | --- |
| 1. *If no:* How could it have been better? Please comment: | |
| 1. Is there any other information that would have been helpful for you? | Yes 🞏 No 🞏 |
| 1. *If yes:* What would that have been? Please comment: | |
| 1. Is there anything that could have prevented you from attending the COPD assessment (such as travel costs, family commitments, etc…). Please comment: | |
| 1. What was helpful for you about the COPD assessment? Please comment: | |
| 1. What else might have been helpful? Please comment: | |
| 1. Did you get enough information about the results of your COPD assessment? | Yes 🞏 No 🞏 |
| 1. *If no:* Please comment: | |
| 1. Overall, how did you feel about your experience of getting a CODP assessment as part of your lung health check? Please comment:  \|  \|  \|  \|  \|  \| \| --- \| --- \| --- \| --- \| --- \| \| I was very happy with the way it went \| It was OK \| Neutral /  Don’t know \| It wasn’t great \| I was unhappy with the way it went \|   Please add any comments you would like to make: | |
| 1. Is there anything else you can tell us about your experience of being assessed for COPD?   Please comment: | |

### Appendix 15: Qualitative interview after COPD assessment

**Interview Schedule**

Meet and greet/whakawhānaungatanga/Karakia

Discuss purpose and reminder re: dates and process they underwent (assessment and screening)

Note: have on hand pictures of tools used as a reminder – playing on the assumption participants may have had multiple medical appointments pre and post timeframe.

Go over information sheet (will be sent prior) and a copy carried with interviewer.

Consent signed (2 x copies – one to be left with participant).

Interview: What was participant and or whānau experience undergoing the LCS and COPD assessment?

1. Tell me about your experience in relation to LCS and COPD assessment
   Go through each step: appointment: being contacted (method), pre and post attendance, understanding process, information, outcome, next steps, did you have to take time off work, what motivated you to participate?
   1. What worked well, what did they like, what could have been better/more helpful?
   2. What did not work well for them, what were some of the challenges or difficulties through each step? (e.g. understanding, location, parking etc)
   3. Do you have any feedback on the screening/assessment (if necessary, go through the process including: arrival, workforce, attitude, way you treated, process, equipment, feelings and information post screening)?
   4. If you knew someone else had the same appointment, what would you tell them to expect or what would you say that might be helpful or make things easier for them?
2. Was it helpful having both the COPD assessment and screening at the same appointment? If so or not, can you tell me why? Is that something that would be good to do in the future for others?
3. Can you tell me how you felt after the assessment?
   1. How were findings given, support offered or present, next steps and information) is there anything that could have been done better or differently in relation to being told the outcome/findings
4. Tell me what it was like for you to be part of the shared decision-making COPD results management process (lay terms)
   1. Reminding them of what some of the decisions and results may have been and what the next steps were...
5. Do you know if information goes to your GP? Query the experience of provision of information to their GP
   1. Are you happy with information that goes to your GP, are you comfortable asking about information, do you understand what goes to your GP?
6. Is there anything they want to ask of us, their healthcare professional or the research team, or to add?

Closing / Karakia

### Appendix 16: Survey after Biomarker Assessment (all Biomarker participants)

Tēnā koe

Thank you for taking part in the biomarker assessment for lung cancer screening. We would like to know how you found this. Please complete this survey – this will help us improve the way we do biomarker assessments in the future. This survey is voluntary. (Note – this interview will be conducted with the study nurse, immediately after the assessment).

| 1. Was the biomarker assessment explained to you in a way that you understood? | Yes 🞏 No 🞏 |
| --- | --- |
| 1. *If no:* How could it have been better? Please comment: | |
| 1. Is there any other information that would have been helpful for you? | Yes 🞏 No 🞏 |
| 1. *If yes:* What would that have been? Please comment: | |
| 1. Is there anything that could have prevented you from attending the biomarker assessment appointment (such as travel costs, family commitments, etc…). Please comment: | |
| 1. What was helpful for you about the biomarker assessment? Please comment: | |
| 1. What else might have been helpful? Please comment: | |
| 1. Is there anything else you can tell us about your experience today?   Please comment: | |
| 1. Overall, how did you feel about your experience of getting a biomarker test as part of your lung health check? Please comment:  \|  \|  \|  \|  \|  \| \| --- \| --- \| --- \| --- \| --- \| \| I was very happy with the way it went \| It was OK \| Neutral /  Don’t know \| It wasn’t great \| I was unhappy with the way it went \|   Please add any comments you would like to make: | |

### Appendix 17: Training evaluation survey

Tēnā koe,

In your role as a provider of support to the participants of the Lung Screening Study, you recieved some training about the study, about lung cancer and about how to talk to participants with positive scans and concerning results. We would like to invite you to complete a short questionnaire to provide feedback on this training.

Your individual feedback will not be identifiable. Any report will only include summarised findings, meaning anyone reading the report will not be able to tell who took part in the survey or who said what,

Do I have to take part?

Your participation is voluntary- you do not have to participate if you don't want to. You can also decline to answer any specific questions. Whether or not you agree to take part will have no impact on your employment or on any other aspect of your work.

By answering these questions, you are consenting to the research team including this information in an analysis of how well the lung screening research programme is working. All answers are anonymous and you will not be able to be identified.

| **1)** | **Please indicate your role:** | ****  Practice Nurse  GP  Other  [reset](javascript:;) |
| --- | --- | --- |
| **2)** | Please rate your knowledge of the following **before** you received the study training:  **Lung cancer signs and symptoms** | \| \| 1- very poor \| 5 \| 9- very good \| \| --- \| --- \| --- \| \| \| \| \| --- \| --- \| --- \| --- \| --- \| --- \| \|  \|  \|  \| \| Change the slider above to set a response \| \| \|   [reset](javascript:;) |
| **3)** | **Appropriate referral pathways for people diagnosed with a lung nodule:** | \| \| 1- Very poor \| 5 \| 9- Very good \| \| --- \| --- \| --- \| \| \| \| \| --- \| --- \| --- \| --- \| --- \| --- \| \|  \|  \|  \| \| Change the slider above to set a response \| \| \|   [reset](javascript:;) |
| **4)** | **Before this study, how confident were you dealing with a person with a suspected lung cancer?** | ****No Confidence Uncertain Confident Very Confident  [reset](javascript:;) |
| **5)** | Please rate your knowledge of the following **after**you received the study training:  **Lung cancer signs and symptoms** | \| \| 1- very poor \| 5 \| 9- very good \| \| --- \| --- \| --- \| \| \| \| \| --- \| --- \| --- \| --- \| --- \| --- \| \|  \|  \|  \| \| Change the slider above to set a response \| \| \|   [reset](javascript:;) |
| **6)** | **Appropriate referral pathways for people diagnosed with a lung nodule:** | \| \| 1- Very poor \| 5 \| 9- Very good \| \| --- \| --- \| --- \| \| \| \| \| --- \| --- \| --- \| --- \| --- \| --- \| \|  \|  \|  \| \| Change the slider above to set a response \| \| \|   [reset](javascript:;) |
| **7)** | **After this study, how confident are you dealing with a person with a suspected lung cancer?** | ****No Confidence Uncertain Confident Very Confident  [reset](javascript:;) |
| **8)** | **Please indicate how well you think the training prepared you to support participants of the Lung Screening Study:** | \| \| 1- Very poorly \| 5 \| 9- Very well \| \| --- \| --- \| --- \| \| \| \| \| --- \| --- \| --- \| --- \| --- \| --- \| \|  \|  \|  \| \| Change the slider above to set a response \| \| \|   [reset](javascript:;) |
| **9)** | **Specifically, please indicate how well you think the Shared Decision Making materials prepared you to support participants of the Lung Screening Study:** | \| \| 1- Very poorly \| 5 \| 9- Very well \| \| --- \| --- \| --- \| \| \| \| \| --- \| --- \| --- \| --- \| --- \| --- \| \|  \|  \|  \| \| Change the slider above to set a response \| \| \|   [reset](javascript:;) |
| **10)** | **Please indicate how helpful you found the Shared Decision Making process when explaining the study to potential participants:** | ****Not at all helpful Somewhat helpful Extremely helpful  [reset](javascript:;) |
| **11)** | **Do you have any comments that you would like to make about the training received?** | **** |

### Appendix 18: Requirements for authorship

In terms of future publications, investigators will need to meet the following threshold to be included as an author: They must

- make substantial contributions to the research,
- be accountable for the research as a whole,
- give final approval to the manuscript, and
- contribute to drafting or critically revising the manuscript
